# Role of E-cadherin in epithelial barrier dysfunction: implications for bacterial infection, inflammation, and disease pathogenesis

**DOI:** 10.3389/fcimb.2025.1506636

**Published:** 2025-02-11

**Authors:** Peter Lialios, Stella Alimperti

**Affiliations:** ^1^ Department of Biochemistry and Molecular & Cellular Biology, Georgetown University, Washington, DC, United States; ^2^ Center for Biological and Biomedical Engineering, Georgetown University, Washington, DC, United States

**Keywords:** E-cadherin, epithelial barrier, infection, bacteria, inflammation, homeostasis, disease pathogenesis

## Abstract

Epithelial barriers serve as critical defense lines against microbial infiltration and maintain tissue homeostasis. E-cadherin, an essential component of adherens junctions, has emerged as a pivotal molecule that secures epithelial homeostasis. Lately, its pleiotropic role beyond barrier function, including its involvement in immune responses, has become more evident. Herein, we delve into the intricate relationship between (dys)regulation of epithelial homeostasis and the versatile functionality of E-cadherin, describing complex mechanisms that underlie barrier integrity and disruption in disease pathogenesis such as bacterial infection and inflammation, among others. Clinical implications of E-cadherin perturbations in host pathophysiology are emphasized; downregulation, proteolytic phenomena, abnormal localization/signaling and aberrant immune reactions are linked with a broad spectrum of pathology beyond infectious diseases. Finally, potential therapeutic interventions that may harness E-cadherin to mitigate barrier-associated tissue damage are explored. Overall, this review highlights the crucial role of E-cadherin in systemic health, offering insights that could pave the way for strategies to reinforce/restore barrier integrity and treat related diseases.

## 1 Introduction

The epithelial barrier is essential for maintaining tissue homeostasis and protecting against exogenous insults. Loss of barrier function results in severance of the intricate structural framework of the epithelia and increased susceptibility to noxious stimuli such as bacterial infection and inflammation (Groeger and Meyle, 2015; Rogers et al., 2023). Bacterial pathogens are known to exploit transcytosis, as well as other uptake mechanisms like internalization or paracytosis (intercellular passage), to penetrate epithelial and other tissue barriers. These strategies enable them to reach underlying niches or access the intra- and sub-epithelial spaces, facilitating their spread (Kaper et al., 2004; Edwards and Massey, 2011; Nikitas et al., 2011; Zhu et al., 2024). To this end, it is essential to elucidate cellular phenomena and understand the key regulatory events that govern the integrity of a well-controlled epithelial barrier. This may pave the way for new therapeutic avenues that will enable the development of targeted interventions to mitigate barrier impairment and, ultimately, restore its functionality in pathophysiological conditions.

**Figure 1:**
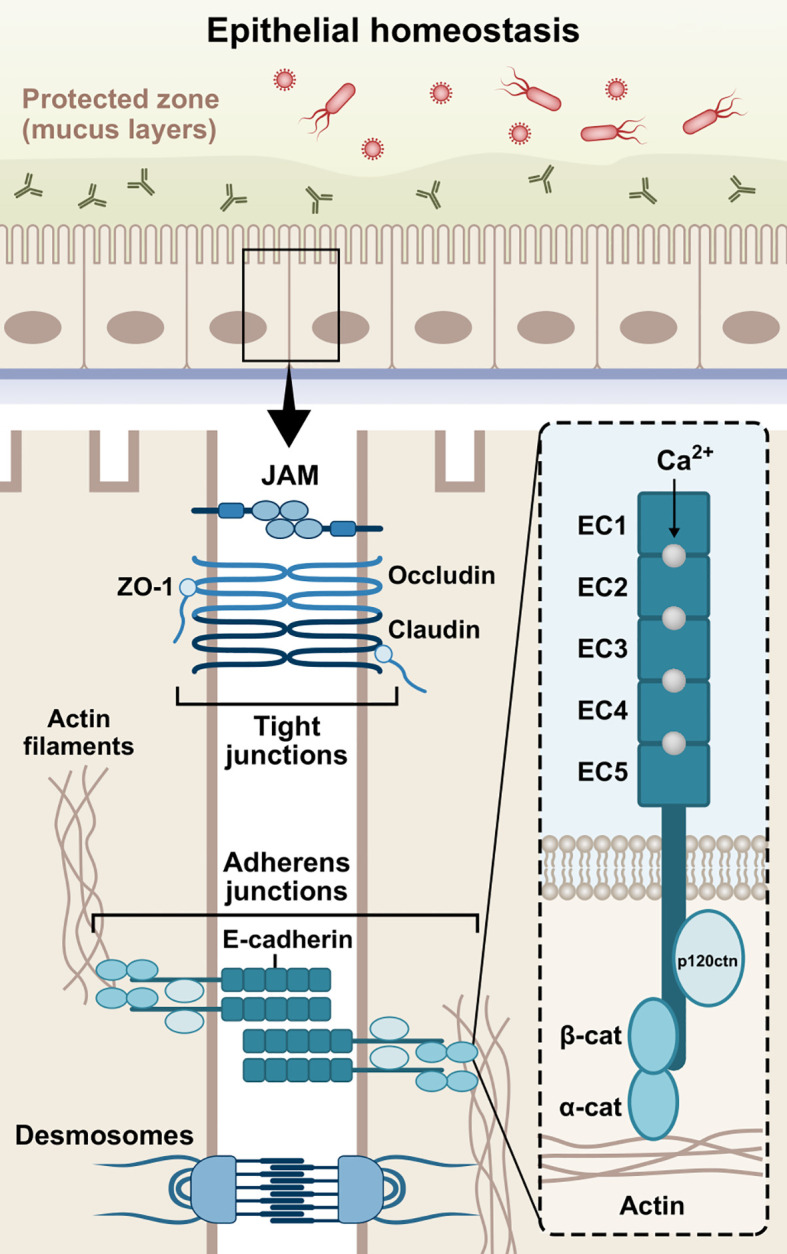
Schematic structure of E-cadherin. The extracellular domain contains five 110 amino acids repeated regions (EC1-EC5), in which the Ca^2+^ ions work as inter-domain linkers to stabilize the adhesive interactions between adjoining cells. The single-pass transmembrane region of E-cadherin transverses the phospholipid bilayer and facilitates the interactions of the extracellular domains with the cytoplasmic domain. The cytoplasmic tail consists of roughly 150 amino acids and regulates downstream signaling pathways. Cadherins initially form cis-dimers on the same cells, followed by the formation of trans-dimers with cadherins on adjacent cells, establishing adhesion across the paracellular space. The three domains are involved in the epithelial barrier function via formation and stabilization of AJs. JAM, junctional adhesion molecule; ZO-1, zonula occludens-1; β-cat, β-catenin; α-cat, α-catenin; p120ctn, p120 catenin; EC1-5, extracellular cadherin repeats 1-5.

The epithelial barrier acts as a physical and immunological barrier, separating the internal milieu from the external environment. It represents a composite network of cell adhesion molecules (CAMs), such as adherens junctions (AJs), tight junctions (TJs), and desmosomes, which collectively maintain the epithelial polarity and barrier microarchitecture (**Figure 1**) (Adil et al., 2021). Infectious agents dissociate these junctional complexes and destabilize the selective permeability and structural coherence, facilitating barrier breach and pathogen invasion into the interstitial tissues (Groeger and Meyle, 2015; Rogers et al., 2023).

The formation of AJs requires the presence of Ca^2+^-dependent transmembrane adhesion glycoproteins, named cadherins. They act more than mere cell glue designated to serve mechanical cohesion between adjacent cells; they orchestrate junctional assembly and inter-junctional communication, and participate in signaling pathways that regulate cellular behavior, such as proliferation, migration, differentiation, epithelial repair, wound healing, or even morphogenesis (Stockinger et al., 2001; Gumbiner, 2005; Halbleib and Nelson, 2006; Van Roy and Berx, 2008, 2008; Van Den Bossche et al., 2012). The most well-studied cadherins are the classical vertebrate cadherins, which have been named based on the tissue in which they are expressed. Neuronal cells mainly express N-cadherin (CDH2), while epithelial cells highly express E-cadherin (CDH1) (Rajwar, 2015; László and Lele, 2022; Kadeh et al., 2023). P-cadherin (CDH3) has been found in breast tissue, skin, and hair follicles, as well as lungs and placenta among others (Vieira and Paredes, 2015). In addition, VE-cadherin (CDH5) is specifically expressed in vascular endothelial cells, where it controls their behavior during angiogenesis (Nan et al., 2023), while K-cadherin (CDH6) is primarily found in the kidney (Cho et al., 1998; Thedieck et al., 2005) and R-cadherin (CDH4) mainly in the brain (Martinez-Garay et al., 2016). Interestingly, it has been found that E-cadherin is also present in immune cells, such as dendritic cells (DCs), macrophages, and T-cells (Riedl et al., 2000; Van Den Bossche et al., 2012; Van Den Bossche and Van Ginderachter, 2013; Charnley et al., 2023; Davies et al., 2024). E-cadherin is a type-I cadherin encoded by the CDH1 gene on chromosome 16q22 (Van Roy and Berx, 2008). The E-cadherin molecule is composed of three distinct structural domains, namely an extracellular domain, consisting of 5 repeated regions (EC1-EC5), which engages in homotypic (cis- and trans-dimers) and heterotypic cell-cell interactions, a single-pass transmembrane domain, and a cytoplasmic tail which regulates downstream signaling (Gumbiner, 2005; Hulpiau and Van Roy, 2009). The domain structure of E-cadherin is illustrated in **Figure 1**.

This review aims to provide a comprehensive overview of E-cadherin as a major junctional molecule with respect to tissue homeostasis and its dysregulation in the etiopathogenesis of bacterial infections, inflammatory, and other conditions. Initially, we report the molecular underpinnings of E-cadherin-directed cell-cell adhesion and relevant signaling pathways in homeostasis. Next, we describe E-cadherin-mediated mechanisms in bacterial infections, inflammation, and other diseases by delving into alterations in E-cadherin expression, localization, and functionality. Furthermore, we highlight the clinical implications of epithelial barrier dysfunction and the mechanistic and immunological involvement of E-cadherin in disease across various tissues, emphasizing numerous infection examples and inflammation models. Lastly, we examine potential therapeutic strategies targeting junctional compounds and E-cadherin to enhance and restore epithelial barrier integrity and tackle infection.

## 2 Homeostatic regulation of the epithelial barrier via E-cadherin

**Figure 2:**
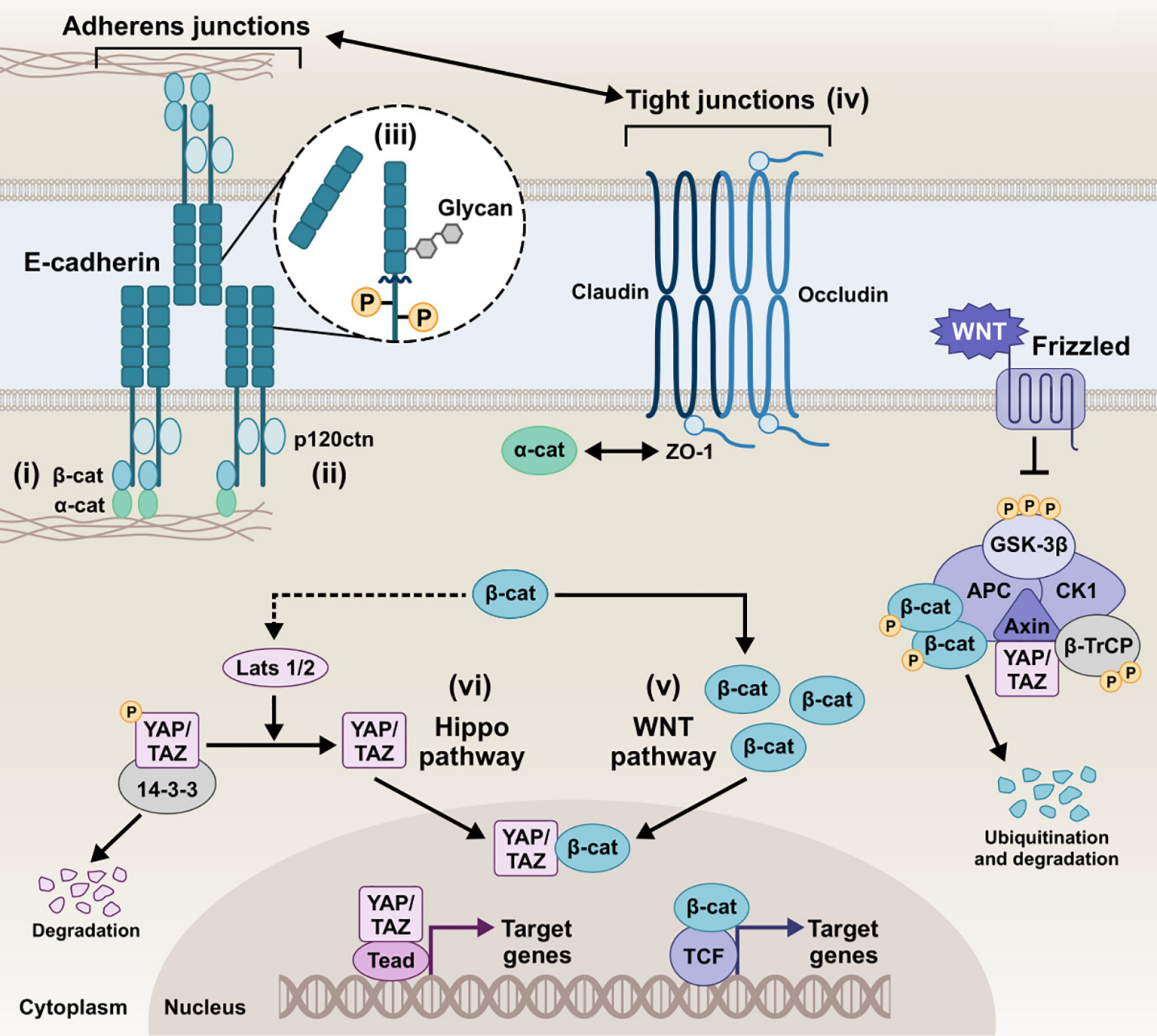
Schematic representation of the E-cadherin interactions and homeostatic mechanisms involved in the regulation of the epithelial barrier of epithelial barrier. i) E-cadherin/β-catenin/actin complex, ii) E-cadherin/p120ctn complex, iii) E-cadherin post-translational modifications, iv) Tight junctions and E-cadherin, v) E-cadherin and Wnt pathways, vi) E-cadherin and Hippo pathway, collectively play a critical role in tightly regulating cellular behavior and intercellular communication. Intricate modulation of the AJs integrity, downstream signaling, and overall epithelial barrier function preserves homeostatic conditions in the host tissue. β-cat, β-catenin; α-catenin, α-catenin; p120ctn, p120 catenin; ZO-1, zonula occludens 1; GSK-3β, glycogen synthase kinase-3 beta; CK1, casein kinase 1; APC, anaphase-promoting complex; β-TrCP, beta-transducin repeats-containing protein; YAP, yes-associated protein; TAZ, transcriptional co-activator with PDZ-binding motif; LATS1/2, large tumor suppressor kinase 1/2; Tead, transcriptional enhanced associate domain; TCF, T-cell factor.

E-cadherin plays a vital role in tissue homeostasis by contributing to selective, semi-permeable barrier structure features via sealing the intercellular spaces between the cells and promoting the formation of AJs (Wheelock and Johnson, 2003; Takeichi, 2014). Herein, we aim to report E-cadherin-mediated mechanisms that are involved in the barrier assembly and are responsible for maintaining epithelial homeostasis (**Figure 2**).

### 2.1 E-cadherin/β-catenin/actin complex

The canonical pathways involved in AJ assembly demonstrated the E-cadherin clustering controlled by the intracellular tail and the coupled actin cytoskeleton (Yap et al., 1998; Wu et al., 2015; Biswas and Zaidel-Bar, 2017). Specifically, the C-terminus of the intracellular tail interacts with a group of adaptor proteins called armadillo catenins, namely β-catenin and plakoglobin (γ-catenin), which anchor E-cadherin to the peri-junctional actin cytoskeleton. γ-Catenin is primarily localized at desmosomes and AJs, interacting with desmogleins/desmocollins and cadherins, respectively, and can compensate for β-catenin loss at AJs without disrupting desmosomal integrity (Diane Wickline et al., 2013). The E-cadherin-catenin complex -known as CCC- is composed of β-catenin (or plakoglobin), which directly tethers via its central Armadillo domain to the cytosolic tail of E-cadherin and via the N-terminal domain to α-catenin, which in turn links the compound to the actin filaments (F-actin) (Pećina-Šlaus, 2003; Kobielak and Fuchs, 2004; Hulpiau and Van Roy, 2009). The binding of α-catenin to F-actin requires α-catenin homodimers, whereas α-catenin binds to E-cadherin/β-catenin complex in its monomeric form. EPLIN (i.e., epithelial protein lost in neoplasm) represents the missing link between the CCC and the apical circumferential actin belt, coupling cortical actin filament bundles to the monomeric α-catenin of the assembly (Abe and Takeichi, 2008).

### 2.2 E-cadherin/p120ctn complex

A highly conserved sequence in the juxtamembrane domain of E-cadherin is responsible for coupling with another catenin, named p120 catenin (p120ctn), whose binding is fundamental for the AJ assembly (Thoreson et al., 2000; Van Roy and Berx, 2008). p120ctn acts as a master regulator of E-cadherin’s cell surface delivery and functional integrity by inhibiting internalization pathways that promote E-cadherin degradation and facilitating plasma membrane recycling (Davis et al., 2003). It has been reported that the juxtamembrane region primarily mediates the lateral clustering of cadherin molecules, further reinforcing the role of p120ctn as a key contributor to cluster formation and adhesion strengthening (Yap et al., 1998). Moreover, p120ctn is an important mediator for the Rho-associated protein kinase (ROCK)/E-cadherin interaction. ROCK is a serine-threonine kinase involved in the regulation of cadherin function. Constitutive activation of ROCK leads to disruption of AJs, whereas pharmacological inhibition of ROCK promotes AJ stability (Wójciak-Stothard et al., 2001; Grothaus et al., 2018).

### 2.3 Post-translational events

Post-translational processing of E-cadherin, most prominently including phosphorylation, O-glycosylation, N-glycosylation, and proteolytic cleavage, has been extensively described to dictate its function and redistribution dynamics. Serine phosphorylation of the β-catenin-binding domain, for instance, has been reported to be constitutive to cadherin-catenin complex formation and stabilization by increasing β-catenin binding affinity and regulating E-cadherin’s biosynthesis and trafficking (McEwen et al., 2014). Effector phosphorylation of p120ctn and β-catenin also seem to -inversely- contribute to the E-cadherin/catenin association and partly control E-cadherin’s surface stability (Roura et al., 1999; Fukumoto et al., 2008). Cytoplasmic O-glycosylation (O-GlcNAc) of newly synthesized E-cadherin regulates its secretory path, causing retention in the endoplasmic reticulum and cell surface transit arrest. In its absence, unimpeded export to the membrane delays apoptosis and rescues E-cadherin recruitment to adhesion sites (Geng et al., 2012). Ectodomain N-glycosylation constitutes the most prevalent post-translational modification, boasting four potential sites (two in EC4 and two in EC5) in the extracellular domain of human E-cadherin. In addition to E-cadherin folding and trafficking, N-glycan remodeling can be instrumental to functional junction organization, with the extent of N-glycan branching/complexity negatively associating with adhesive strength (Pinho et al., 2011). Another functionally-impairing post-translational event E-cadherin can undergo is proteolytic truncation by endogenous proteases, which more prominently results in the release of soluble E-cadherin (sE-cad) fragments, as discussed in more detail below. sE-cad is approximately 80 kDa in size, generated by α-secretase cleavage on the extracellular face of the plasma membrane, which is catalyzed by various proteases, including matrix metalloproteinases (MMPs), members of a disintegrin and metalloproteinase (ADAMs) family, plasmin, and kallikrein 7 (David and Rajasekaran, 2012). The shed sE-cad fragment can diffuse into the extracellular environment, where it retains the ability to form homophilic bonds and pair with intact, full-length molecules, interfering with the function of adhesion-competent E-cadherin. Moreover, it can chemotactically anchor E-cadherin on migrating cells and upregulate MMPs, thereby further destabilizing epithelial integrity (Samuels et al., 2023). Ectodomain shedding disrupts the intact E-cadherin junctional complexes, with circulating sE-cad harboring biological effect amplification in the context of proliferative and survival/apoptotic resistance signals, migratory and invasive abilities due to loss of barrier function, inflammation, and tumorigenesis (Grabowska, 2012). The remaining membrane-bound C-terminal fragment of E-cadherin (38 kDa, E-cad/CTF1) can then be cleaved by a γ-secretase/presenilin-1/2, injecting a 33-kDa E-cad/CTF2 fragment into the cytosol. This unleashes β-catenin which can promote the oncogenic canonical Wnt pathway, with E-cadherin sheddase matrilysin (MMP-7) among the transcriptional targets. Also, p120ctn remains E-cadherin-bound and can mediate E-cad/CTF2 translocation to the nucleus and subsequent DNA binding, where E-cad/CTF2 modulates p120ctn-Kaiso-mediated pathway to suppress apoptosis (Ferber et al., 2008). In addition to fragmentation into CTF1 and CTF2, generation of a 29kDa E-cad/CTF3 by caspase-3 has been observed in apoptosis and cancer progression (Craig and Brady-Kalnay, 2011; Yang et al., 2017).

### 2.4 E-cadherin and tight junctions (TJs)

Tungal et al. demonstrated that E-cadherin is crucial for maintaining epithelial barrier function *in vivo* by regulating TJ formation and stability. Specifically, E-cadherin coordinates the trafficking and positioning of TJ proteins, facilitating the localized integration of key molecules such as the cytoplasmic scaffolding zonula occludens 1 (ZO-1) and claudins, a family of integral membrane proteins that form TJs (Tunggal et al., 2005; Maiers et al., 2013). The communication between AJs, mediated by E-cadherin, and TJs plays a vital role in establishing inter-junctional co-dependence and directing the initial architecture of the epithelial barrier (Ando-Akatsuka et al., 1999; Lázaro et al., 2002; Tunggal et al., 2005).

The functional coupling of AJs and TJs is essential for the maturation of AJs and the early development of TJs. Early studies found that ZO-1 mobilization to the plasma membrane is mediated by catenins, enabling ZO-1 to co-distribute in areas segregated by E-cadherin (Rajasekaran et al., 1996). ZO-1, a key marker of TJs, is closely associated with AJs and the cadherin-catenin complex, transiently binding with α-catenin in nascent junctions (Maiers et al., 2013; Campbell et al., 2017). Knockdown of E-cadherin using siRNA has been shown to reduce ZO-1 expression and lower epithelial resistance in bronchial epithelial cells (Heijink et al., 2010). Additionally, loss of E-cadherin disrupts the organization of ZO-1 and F-actin, as E-cadherin-dependent mechanical circuits play a role in integrating force transduction and signaling pathways that drive junctional polarization necessary for functional epithelial barrier formation (Rübsam et al., 2017).

E-cadherin also regulates epidermal growth factor receptor (EGFR) activity and junctional tension to inhibit premature TJ complex formation in lower layers, while promoting TJ stability and cortical stiffness in apical layers. In E-cadherin knockout models, occludin—a transmembrane protein essential for TJs—and its cytosolic connector ZO-1 exhibit a more punctate or discontinuous pattern at cellular interfaces, explaining why TJ barrier function is compromised in the absence of E-cadherin (Rübsam et al., 2017).

Moreover, TJ proteins can influence E-cadherin regulation. For instance, introducing mutated ZO-1 into a ZO-null cell line inhibits the maturation of AJs during epithelial polarization (Ikenouchi et al., 2007). Additionally, overexpression of claudin-1 has been shown to drive the transcriptional downregulation of E-cadherin through the transcriptional repressor ZEB-1 (Singh et al., 2011). In contrast, overexpression of claudin-7 upregulates E-cadherin expression and enhances cell-cell adhesion, whereas E-cadherin expression does not appear to induce an increase in claudin-7 (Lioni et al., 2007).

### 2.5 E-cadherin and Wnt pathways

The Wnt signaling pathways are evolutionarily conserved cellular communication networks that play a key role in both normal physiological and disease states. Several studies have reported that Wnt signaling governs processes such as cell fate determination, differentiation, proliferation, migration, and polarity. The pathway is divided into two main branches: the canonical Wnt/β-catenin pathway, which involves the stabilization and nuclear translocation of β-catenin, and the non-canonical Wnt pathways, such as the planar cell polarity (PCP) pathway, which operate independently of β-catenin (Komiya and Habas, 2008; Katoh, 2017; Flores-Hernández et al., 2020). Of note, E-cadherin/β-catenin membranous interaction and colocalization sequesters β-catenin to the membrane, inhibiting Wnt activation and epithelial-to-mesenchymal transition (EMT) by averting nuclear translocation of β-catenin. The Wnt/β-catenin signaling culminates in the nucleus with the formation of the TCF/LEF complex, initiating the transcription of Wnt target genes. Loss of E-cadherin results in downregulation of membrane β-catenin binding, whereas nuclear mutant β-catenin induces EMT, dysregulating the assembly of TJs and AJs (Kim et al., 2019). Also, E-cadherin/β-catenin interaction maintains low levels of cytoplasmic β-catenin fraction by inhibiting Wnt signaling (Stockinger et al., 2001). In reverse, the absence of Wnt stimulus empowers β-catenin phosphorylation by a destruction complex consisting of APC, Axin, GSK3β, and CK1, which marks β-catenin for degradation by the proteasome (Stamos and Weis, 2013). β-catenin´s growth-inducing transcriptional activity can thus be counteracted by E-cadherin, which in turn induces cell cycle arrest or, more pronouncedly, apoptosis (Stockinger et al., 2001).

### 2.6 E-cadherin and Hippo pathway

The Hippo pathway is another evolutionarily conserved signaling network that regulates cell-cell communication and tissue homeostasis across species. It integrates environmental signals, including cellular polarity, contact inhibition, soluble factors, and mechanical stimuli, to regulate key biological processes such as cell proliferation, organ/tissue size, development, and regeneration (Cheng et al., 2020; Ahmad et al., 2022; Fu et al., 2022; Nita and Moroishi, 2024; Zhong et al., 2024). It primarily regulates the phosphorylation of Yes-associated protein (YAP) and transcriptional co-activator with PDZ-binding motif (TAZ) by LATS1/2 kinases at multiple serine residues. This phosphorylation facilitates the binding of 14-3-3 proteins, resulting in the retention of YAP/TAZ in the cytoplasm, preventing their nuclear translocation and transcriptional activity, and potentially leading to their proteolytic degradation in the cytosol (Cheng et al., 2020; Zhong et al., 2024). Upon LATS1/2 inactivation, unphosphorylated YAP/TAZ translocate to the nucleus, where it functions as a transcriptional co-activator by associating with the transcriptional enhanced associate domain (TEAD) transcription factor family (Kaan et al., 2017; He et al., 2021). The resulting YAP/TAZ-TEAD complex facilitates the transcriptional activation of numerous target genes, including those encoding critical junctional proteins such as desmogleins and E-cadherin. Inhibition of YAP–TEAD interactions lead to a substantial decrease in both YAP and phospho-YAP levels, significantly impairing cell–cell junction integrity and resulting in the disassembly of AJs and desmosomes (Ahmad et al., 2022). Kim et al. demonstrated that cell-cell adhesion, mediated by homophilic binding of E-cadherin, contributes to YAP inactivation (Kim et al., 2011). Perturbing the E-cadherin/α-catenin complex reduces YAP phosphorylation and increases YAP nuclear accumulation and activity (Kim et al., 2011; Lamar et al., 2012). Studies have shown that the regulation of Hippo pathway kinases and the sequestration of YAP occur at AJs, where several Hippo pathway components are localized (Pan et al., 2018; Ma et al., 2020; Ahmad et al., 2022).

Several studies have also established a connection between Hippo signaling and cell-cell contact through the regulation of TJs, including ZO proteins (Kaan et al., 2017; Ahmad et al., 2022; Guo et al., 2022). Specifically, AMOTL2, a member of the Angiomotin (AMOT) family of proteins, binds directly to the WW domains of YAP via its PPxY motifs, sequestering YAP at TJs and preventing its nuclear activity. In addition, it has been shown that AMOTL2 interacts with LATS2, permitting the recruitment of upstream Hippo components, such as SAV1, to the junctional complex. The interaction between AMOTL2 and LATS2 also facilitates LATS2-mediated YAP phosphorylation, cytoplasmic retention, and inactivation (Paramasivam et al., 2011; Zhao et al., 2011). Intriguingly, the scaffolding functions of AMOTL2 have been described beyond YAP and LATS2, including multiple other junctional proteins like ZO-1 and β-catenin, thus contributing to maintenance of TJ integrity and epithelial polarity (Zhao et al., 2011; Kim et al., 2021). Hippo and canonical Wnt have been reported to engage in crosstalk, particularly through the YAP effector; YAP/TAZ has been described as part of the β-catenin destruction complex and can modulate the Wnt/β-catenin response and β-catenin degradation; in Wnt-OFF cells, YAP/TAZ cytoplasmic sequestration as part of the destruction complex, inhibits Wnt/β-catenin signaling in the cytoplasm. Conversely, in nucleus, YAP/TAZ can contribute to β-catenin-mediated transactivation of genes, with the two co-activators complexing and β-catenin/YAP/TAZ/TEAD co-regulating target genes. Finally, YAP can be a Wnt/β-catenin target gene, with its expression being a driver of proliferation in cancer cells (Konsavage and Yochum, 2013; Sileo et al., 2022).

## 3 E-cadherin regulation in bacterial infections

**Figure 3:**
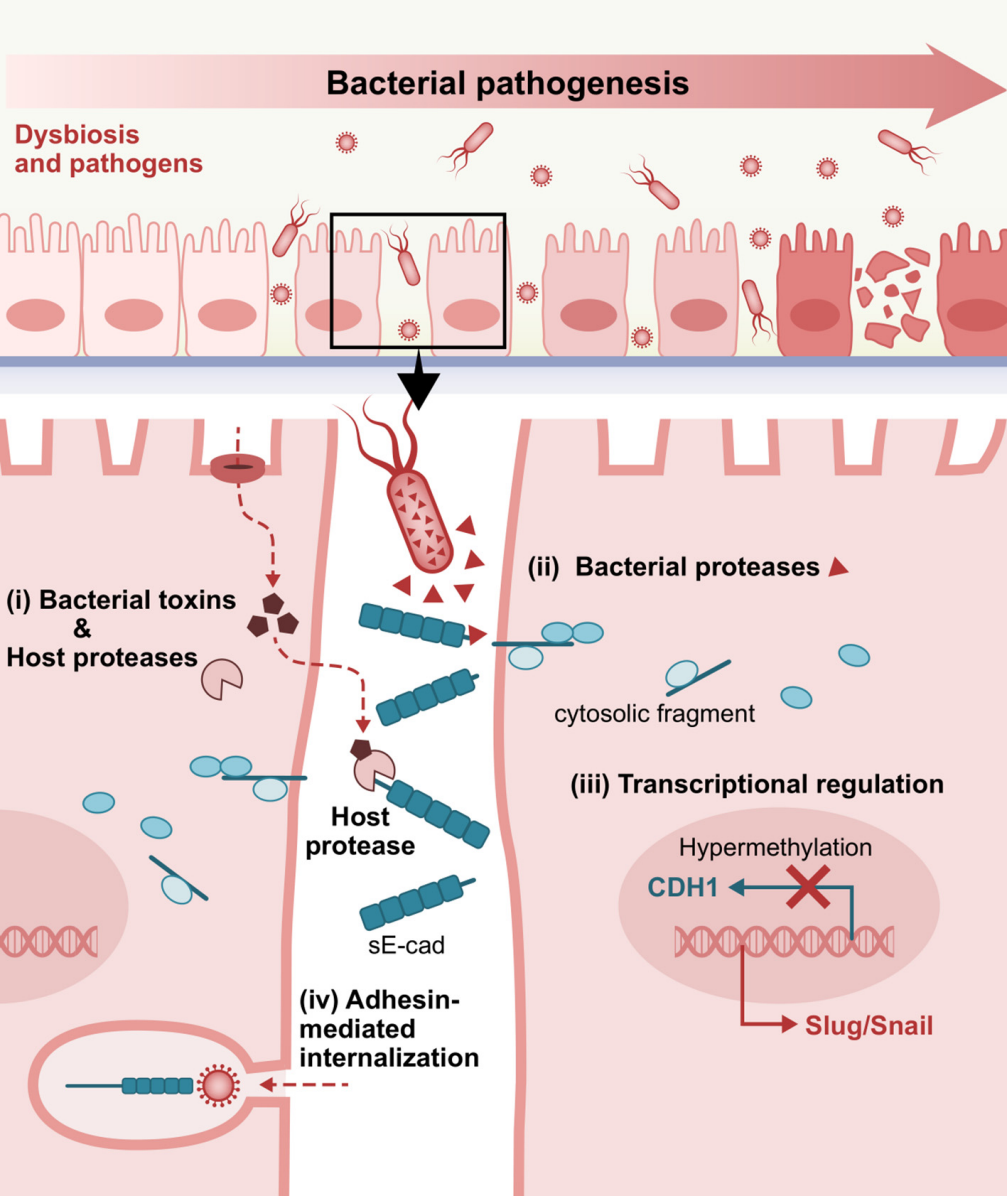
Representative E-cadherin-mediated intercellular interactions that are involved in bacterial pathogenesis. i) Bacterial toxins inducing host proteases, ii) Bacteria-secreted proteases, iii) Dysregulated E-cadherin expression and signaling, and iv) Adhesin-mediated internalization via interactions with the extracellular domain of E-cadherin as a target receptor, play a crucial role in bacterial attachment, invasion into the underlying tissues and consequent establishment and dissemination of infection. sE-cad, soluble E-cadherin fragment.

E-cadherin is considered the gatekeeper of the epithelial barrier, which stands at the frontline of mechanical and immune defense against pathogens. Given the biological complexity of inflammation in epithelial tissues and the range of its clinical manifestations, mucosae and other membranes play a crucial role as the first line of defense against bacterial invasion (Haq et al., 2019; Yang and Yan, 2021; Chegini et al., 2023). Specifically, E-cadherin has been implicated in microbial invasion and dissemination during infectious diseases which breach the epithelial barrier. Herein, we report the direct E-cadherin-driven interactions with infectious agents (**Tables 1**, **2**) as well as pathogen-induced signaling and expression dysregulation, which are involved in the etiopathogenesis of bacterial infections (**Figure 3**).

**Table 1:** Major pathogens, secreted proteases and host sheddases induced by bacterial infection, allow proteolytic degradation of E-cadherin, disruption of the epithelial barrier, and ultimately bacterial invasion and dissemination.

Mechanism	Pathway	Pathogen	E-cadherin Effects or Interactions	References
**(i) Bacterial Toxins and Host Proteases**	**ADAM-mediated pathways**	***Helicobacter pylori***	E-cadherin cleavage and ectodomain shedding, induced calpain-mediated cleavage, elevated sE-cad levels, α-catenin-E-cadherin interaction disruption	(Weydig et al., 2007; Schirrmeister et al., 2009; O’Connor et al., 2011)
		***Pseudomonas aeruginosa***	ADAM10-mediated E-cadherin shedding via toxins, **ExoA**-stimulated calcium ion conduit, **ExlA** activating ADAM10	(Reboud et al., 2017; Aljohmani et al., 2022)
		***Serratia* spp.**	**ShlA** activating ADAM10 and E-cadherin cleavage	(Reboud et al., 2017)
		***Staphylococcus aureus***	**Hla** activating ADAM10 and E-cadherin cleavage	(Inoshima et al., 2011; Von Hoven et al., 2016)
		***Clostridium perfringens***	ADAM10-promoted E-cadherin loss, increased permeability, intracellular vesicles containing digested E-cadherin	(Seike et al., 2019)
	**MMP-mediated pathways**	***Helicobacter pylori***	Upregulation of MMP-9 and MMP-7, E-cadherin ectodomain shedding, EMT induction, MMP-7 induction via RhoA and NF-κB activation	(Noë et al., 2001; Gooz, 2003; Wroblewski et al., 2003; Bergin et al., 2004; McCaig et al., 2006; Kubben et al., 2007; Lee et al., 2007; Symowicz et al., 2007; Yin et al., 2010)
		***Leptospira* spp.**	**LRR20** interacting with E-cadherin, activating MMP-7, degradation of cell-surface E-cadherin, promoting NF-κB pathway activation	(Hsu et al., 2021)
		***Pseudomonas aeruginosa***	High MMP-9 expression and enzyme activity in infected cornea	(McClellan et al., 2006)
		***Staphylococcus aureus***	Upregulated MMP-9 and MMP-7 in nasal mucosa, mid-ear epithelia, and during septic arthritis	(Gjertsson et al., 2005; Wang et al., 2010; Park et al., 2012; Tsai et al., 2018)
		***Streptococcus pneumoniae***	**PLY**-driven E-cadherin cleavage, PMN recruitment, bacterial translocation, complete ablation of E-cadherin by **PFO** or **ILY**	(Xu et al., 2023)
		***Chlamydia* spp.*, Porphyromonas gingivalis***	Excess MMP-9 activity	(Ault et al., 2002; Jotwani et al., 2010; Paolillo et al., 2012)
		***Coxiella burnetii***	Augmented MMP-7 and MMP-9 production, higher sE-cad levels in sera	(Krajinović et al., 2012; Jansen et al., 2017; Mezouar et al., 2019)
	**Miscellaneous Host Proteases**	***Staphylococcus aureus***	**Calpain**-mediated E-cadherin cleavage, cytoskeleton disorganization via RhoA/ROCK/MLC, **Spa** mediates the pathogen’s paracellular penetration	(Soong et al., 2011)
		***Streptococcus oralis, Candida albicans***	**Calpain**-mediated E-cadherin cleavage, synergistic effect promoting systemic dissemination and biofilm formation	(Xu et al., 2016)
		***Helicobacter pylori***	**Caspase-3**-mediated E-cadherin degradation into intracellular fragments, apoptosis induction	(Yang et al., 2017)
		***Pseudomonas aeruginosa, Streptococcus pneumoniae***	**NE**-mediated E-cadherin proteolysis and collateral tissue damage due to excessively activated neutrophils	(Benabid et al., 2012; Boxio et al., 2016; Domon et al., 2018; Domon and Terao, 2021)
**(ii) Bacterial Proteases**	**HtrA**	***Helicobacter pylori, Campylobacter jejuni, enteropathogenic Escherichia coli (EPEC), Shigella flexneri, Salmonella enterica, Yersinia enterocolitica, Proteus mirabilis, Chlamydia* spp.*, Listeria monocytogenes, Bacillus anthracis, Coxiella burnetii, Borrelia burgdorferi, Glaesserella (Haemophilus) parasuis and Actinobacillus pleuropneumoniae***	E-cadherin cleavage of NTF, CTF1 and CTF2 fragments release, promoting pathogen translocation, co-translocation of commensal microbiota, CagA injection, and tyrosine phosphorylation, elevated sE-cad levels, M2-polarized macrophages and downregulation of E-cadherin expression, ECM protein and E-cadherin degradation	(Hoy et al., 2010, 2012; Wu et al., 2011; Boehm et al., 2012; Russell et al., 2013; Abfalter et al., 2016; Elmi et al., 2016; Schmidt et al., 2016a, b; Israeli et al., 2019; Mezouar et al., 2019; Cao et al., 2021; Radhakrishnan et al., 2021; Sharafutdinov et al., 2022, 2024; Zhang et al., 2022; Osman et al., 2023; Canadas-Ortega et al., 2024)
	**BFT or fragilysin**	***Bacteroides fragilis***	E-cadherin step-wise cleavage, β-catenin cytoplasmic translocation and NF-κB activation, IL-8 secretion	(Wu et al., 2007; Rhee et al., 2009; Shiryaev et al., 2014; Choi et al., 2016; Zakharzhevskaya et al., 2017; Pierce et al., 2021; Lee et al., 2022)
	**GelE**	***Enterococcus faecalis***	E-cadherin extracellular domain loss, barrier breakage, colitis development, PAR2 activation	(Steck et al., 2011; Maharshak et al., 2015)
	**Gingipains**	***Porphyromonas gingivalis***	E-cadherin breakdown, host proteins’ proteolytic activation, non-canonical β-catenin activation, peri-implant disease involvement, colitis exacerbation	(Katz et al., 2002; Inaba et al., 2014; Zhou et al., 2015; Hočevar et al., 2018; Eick et al., 2019; Tsuzuno et al., 2021)
	**Miscellaneous Bacterial Proteases**	***Clostridium perfringens***	Cysteine protease-induced E-cadherin degradation	(Pruteanu and Shanahan, 2013)
		***Mycobacterium tuberculosis***	Extracellular serine protease **Rv2569c** mediating E-cadherin cleavage, respiratory epithelial barrier translocation, pathological damage to pulmonary tissues	(Zang et al., 2024)
		***Leptospira interrogans***	E-cadherin displacement, cytoskeletal rearrangement, AJ disassembly, UPS hijacking	(Tokumon et al., 2023)
		**Spontaneous bacterial peritonitis-causing bacteria (*E. coli, P. mirabilis*)**	E-cadherin cleavage by novel protease, TJ protein occludin reduction by enhanced proteosomal activity	(Haderer et al., 2022)

Pathogenic mechanisms, pathogens, key proteases, and toxins are indicated in bold. ADAM, A-disintegrin and metalloproteinase; sE-cad, soluble E-cadherin fragment; ExoA, exotoxin A; ExlA, exolysin; ShlA, pore-forming toxin of Serratia marcescens; Hla, α-hemolysin; MMP, matrix metalloproteinase; RhoA, Ras homolog gene family member A; NF-κB, nuclear factor kappa B; LRR20, leptospira leucine-rich repeat 20; PLY, pneumolysin; PMN, polymorphonuclear neutrophil; PFO, perfringolysin O; ILY, intermedilysin; ROCK, Rho-associated protein kinase; MLC, myosin light chain; Spa, S. aureus protein A; NE, neutrophil elastase; NTF, amino-terminal fragment; CTF, carboxy-terminal fragment; CagA, cytotoxin-associated gene A; ECM, extracellular matrix; IL-8, interleukin-8; PAR2, protease-activated receptor 2; AJ, adherens junction; UPS, ubiquitin-proteasomal system; TJ, tight junction.

**Table 2:** Bacterial mechanisms employing E-cadherin as a target receptor for bacterial attachment and entry.

Pathogen	Interaction with E-cadherin	Mechanism/Effect	References
***L. monocytogenes***	**lnlA** binds to N-terminal EC1 domain	- Initiates “zipper”-like mechanism for entry into epithelial cells - Requires calcium and induces post-translational modifications of E-cadherin - Leads to caveolin-dependent clustering and clathrin-mediated internalization - Also uses InlB for enhanced invasion	(Mengaud et al., 1996; Schubert et al., 2002; Lecuit et al., 2004; Bonazzi et al., 2008; Pentecost et al., 2010; Nikitas et al., 2011; Dellafiora et al., 2020)
***S. pneumoniae***	**PsaA** binds to E-cadherin	- Calcium-dependent binding - Both human and mouse E-cadherin inhibits PsaA-coated adherence to NP cells	(Anderton et al., 2007)
***EPEC***	E-cadherin is recruited at intercellular junctions and interacts with intimin (bacteria) – Tir (host cells) receptor complex	- E-cadherin influences EPEC attachment post initial intimin-Tir interaction - Absence of E-cadherin reduces EPEC adhesiveness	(Login et al., 2018)
***F. nucleatum***	**FadA** binds to EC5 domain	- Promotes attachment and invasion in CRC and non-CRC cells - Induces β-catenin signaling and oncogenic pathways in CRC cells - Affects inflammatory responses based on β-catenin expression	(Rubinstein et al., 2013; Ma et al., 2018)
***C. botulinum***	**Hemagglutinin** binds to EC1-EC2 residues	- Disrupts E-cadherin function by blocking trans-dimerization	(Sugawara et al., 2010; Lee et al., 2014)
***H. pylori***	**CagA** interacts with E-cadherin	- Impairs E-cadherin/β-catenin complex assembly - Leads to β-catenin accumulation and activation of signaling pathways - Interacts with c-Met and p120ctn affecting invasiveness	(Murata-Kamiya et al., 2007; Oliveira et al., 2009)
***C. difficile***	E-cadherin serves as an adherence receptor for spores	- Requires TcdA and TcdB toxins to open AJs and make E-cadherin accessible for spore tethering	(Castro-Córdova et al., 2023)
***L. interrogans***	**Protein Lsa16** and leptospiral lipoproteins (**LIC11711, LIC12587**) bind to E-cadherin	- Allows bacterial attachment to epithelial cells - Induces E-cadherin/β-catenin and NF-κB signaling affecting E-cadherin regulation and *Leptospira* adhesion - E-cadherin downregulation potentially decreases *Leptospira* colonization	(Evangelista et al., 2014; Pereira et al., 2017; Kochi et al., 2019; Hsu et al., 2021)

Pathogens and main adhesins that interact with E-cadherin are indicated in bold.

PsaA, pneumococcal surface adhesin A; NP, nasopharyngeal; EPEC, enteropathogenic Escherichia coli; Tir, translocated intimin receptor; FadA, protein adhesion A; CRC, colorectal cancer; CagA, cytotoxin-associated gene A; c-Met; mesenchymal-epithelial transition factor; p120ctn, p120 catenin; TcdA/TcdB, Clostridioides difficile toxin A/B; AJs, adherens junctions; NF-κB; nuclear factor kappa B.

### 3.1 Bacterial toxins and pathogen-induced host proteases

E-cadherin cleavage to an 80 kDa soluble fragment is one of the primary mechanisms known to provoke functional loss of E-cadherin. The cleavage of E-cadherin is more commonly attributed to matrix metalloproteinases, including MMP-3 (stromelysin-1), MMP-7 (matrilysin), MMP-9 (gelatinase B or gelatinase type IV), as well as certain ADAMs such as ADAM10 (adamalysin) (Noë et al., 2001; David and Rajasekaran, 2012; Boukhedouni et al., 2020; Tao et al., 2021; Im et al., 2022).

#### 3.1.1 ADAM-mediated pathways

Elevated sE-cad levels have been reported in sera of *Helicobacter pylori (H. pylori)*-positive patients (O’Connor et al., 2011). *H. pylori* infection, the causative agent of peptic ulcers and one of the leading risk factors of gastric cancer, was found to trigger significant E-cadherin ectodomain shedding, potentially employing host’s native sheddases, such as ADAM10 or less pronouncedly, ADAM19, as executors (Schirrmeister et al., 2009). Loss of full-length E-cadherin can occur irrespective of *H.pylori* virulence factor CagA and without transactivating β-catenin transcriptional signaling, while disassembly of AJ complexes rapidly follows disruption of α-catenin-E-cadherin interaction and subsequent disassembly of the E-cadherin/β-catenin/p120ctn complex from the actin cytoskeleton (Weydig et al., 2007). *H. pylori* can also induce calpain-mediated cleavage, resulting in the production of a 100 kDa truncated E-cadherin form, independent of CagA and VacA, but rather via activation of TLR2 by a putative proteinaceous *H. pylori* surface component. Cytoplasmic translocation of β-catenin and internalization of E-cadherin ensues, with intracellular redistribution of E-cadherin away from cell-contact sites (O’Connor et al., 2011).

*Pseudomonas aeruginosa (P. aeruginosa*) infection was recently shown to modulate epithelial permeability by triggering exosomal ADAM10-mediated E-cadherin shedding activity via its secreted toxin repertoire and an Exotoxin A (ExoA)-stimulated calcium ion conduit intracellularly (Aljohmani et al., 2022). Likewise, other pore-forming toxins, such as *P. aeruginosa*-derived exolysin (ExlA), *Serratia marcescens*-derived ShlA, and *Staphylococcus aureus* α-toxin or α-hemolysin (Hla) were also found to drive ADAM10 activation and subsequent cadherin cleavage, through potentiating calcium influx and cell death (Inoshima et al., 2011; Von Hoven et al., 2016; Reboud et al., 2017). In the case of *Serratia* infection, it has been reported that *S. proteamaculans* invasion requires full-length E-cadherin, while *S. grimesii* invasiveness can be promoted by both full-length and truncated E-cadherin. Interestingly, E-cadherin expression was shown to increase and redistribute in cell compartments in response to *Serratia* infection (Tsaplina et al., 2023).

*Clostridium perfringens*, which is known to cause food poisoning and gas gangrene, encodes a pore-forming toxin named delta-toxin, which can similarly trigger ADAM10-promoted E-cadherin loss in Caco-2 cells, resulting in increased permeability and fluid accumulation in the ileal loop. With respect to E-cadherin degradation, investigators observed the distribution of digested E-cadherin in intracellular vesicles of shedding cells derived from the damaged intestinal villi as soon as 1h after toxin administration (Seike et al., 2019).

#### 3.1.2 MMP-mediated pathways

A plethora of proteases extending to members of the MMP family, whose substrates include E-cadherin, such as MMP-9 and MMP-7 (matrilysin) (Noë et al., 2001; Lee et al., 2007; Symowicz et al., 2007), are upregulated in *H.pylori*-infected gastric epithelial tissues (Gooz, 2003; Wroblewski et al., 2003; Bergin et al., 2004; McCaig et al., 2006); MMP-9 exhibits 19-fold higher activity in infected gastric mucosae compared to uninfected ones and is secreted by gastric macrophages in response to bacteria, while it decreases significantly upon *H. pylori* eradication (Bergin et al., 2004; Kubben et al., 2007). Adherence of the pathogen induced MMP-7 in AGS cells via RhoA and nuclear factor kappa B (NF-κB) activation (Wroblewski et al., 2003). *H. pylori*-directed EMT through upregulation of E-cadherin-repressive transcription factors Snail and Slug and gastric microenvironment remodeling contribute to its pathogenicity (McCaig et al., 2006; Yin et al., 2010).

A key example of host-pathogen interactions inducing MMP-mediated degradation of E-cadherin is during leptospirosis. An outer membrane virulence factor, leptospira leucine-rich repeat 20 (LRR20), was shown to interact with E-cadherin and mediate its degradation by activating downstream E-cadherin signaling; LRR20 can promote the nuclear translocation of activated β-catenin, significantly increasing MMP-7 expression in a dose and time-dependent manner. LRR20-induced MMP-7 consequently degrades cell-surface E-cadherin, which in turn promotes NF-κB pathway activation (Hsu et al., 2021).

In *P. aeruginosa* keratitis, MMP-9 was reported to show high expression and greater enzyme zymography activity in the infected cornea of susceptible B6 mice versus normal cornea of resistant BALB/c mice (McClellan et al., 2006).

MMP-9 was significantly upregulated by *S. aureus* in infected nasal mucosa and mid-ear epithelia, namely chronic rhinosinusitis and lipoteichoic acid-induced otitis media, respectively (Wang et al., 2010; Park et al., 2012), while *S. aureus*-induced expression depends on PGE_2_/IL-6 during infection-associated aortic inflammation (Tsai et al., 2018). Elevated MMP-7 contributes to *S. aureus* septic arthritis pathogenesis, but interestingly, it also eliminates the increased bacterial burden by enhancing bacterial clearance (Gjertsson et al., 2005).

Pneumolysin’s (PLY) pore-forming activity was shown to be essential for *Streptococcus pneumoniae* to elicit cleavage and subvert organization of E-cadherin at a MOI of 2, though a putatively induced proteolytic executor that remains to be identified (Xu et al., 2023). This low-dose infection drives the recruitment of polymorphonuclear neutrophils (PMNs) and bacterial translocation in a PLY-dependent manner, even in absence of epithelial detachment, while other pore-forming virulence factors of the cholesterol-dependent cytolysins family, such as perfringolysin O (PFO) or intermedilysin (ILY), resulted in almost complete ablation of E-cadherin, indicating a likely pathogenetic mechanism (Xu et al., 2023). Excess MMP-9 activity has been indicated to participate in the pathogenesis of *Chlamydia* spp. and *P. gingivalis* infections (Ault et al., 2002; Jotwani et al., 2010; Paolillo et al., 2012). *Coxiella burnetii*, the etiologic agent of Q fever, can also manifest with augmented MMP(-7,9) production in the acute and persistent form of infection, along with higher sE-cad serum concentrations (Krajinović et al., 2012; Jansen et al., 2017; Mezouar et al., 2019).

#### 3.1.3 Miscellaneous host proteases

Calcium-dependent, non-lysosomal cysteine proteases named calpains, are also known to mediate occludin and E-cadherin cleavage and can be induced by wild-type *S. aureus* in an EGFR-dependent manner. *S. aureus* protein A (Spa) mediates the pathogen’s paracellular penetration into polarized airway epithelial monolayers via tumor necrosis factor (TNF) receptor 1 and EGFR stimulation and consequent RhoA/ROCK/MLC activation that disorganizes cytoskeleton distribution, while calpain activity also facilitates staphylococcal transmigration through the ruptured paracellular junctions (Soong et al., 2011). Augmented calpain-mediated E-cadherin reduction has also been observed as a synergistic effect of *Streptococcus oralis* and *Candida albicans* coinfection, promoting their systemic dissemination and pathogenic potential of their biofilms (Xu et al., 2016).

Caspase-3, a protease “executioner” involved in apoptosis, has also been associated with E-cadherin dismantling. Degradation of full-length E-cadherin into 3 intracellular/carboxy-terminal fragments (CTF1, CTF2, CTF3) by *H. pylori* is reportedly coupled with cleaved-caspase-3 upregulation and induction of gastric epithelial cells’ apoptosis (Yang et al., 2017).

Inflammatory responses triggered during bacterial infections are primarily driven by neutrophils. Neutrophil elastase (NE), a serine protease released by neutrophils at the site of acute lung injury, plays a key role in shaping the proteolytic environment during infections, particularly in PMN-rich pathologies. While NE serves a protective function against pathogens, excessive neutrophil activation and dysregulated NE secretion during bacterial infections can lead to tissue damage. Elevated NE levels have been observed in conditions such as pneumonia caused by *Pseudomonas aeruginosa*, pneumococcal pneumonia, and bacterial exacerbations of chronic obstructive pulmonary disease (COPD) (Benabid et al., 2012; Domon et al., 2018; Thulborn et al., 2019; Domon and Terao, 2021). In a mouse model of *P. aeruginosa* H103 pneumonia, significant amounts of active NE were detected in bronchoalveolar lavage (BAL) fluids, alongside an approximately 80 kDa fragment of E-cadherin, indicative of its degradation in the alveolar space. This effect was observed after eliminating the confounding influence of bacterial metalloelastases, suggesting that NE itself contributes to E-cadherin breakdown (Boxio et al., 2016).

### 3.2 Bacterial proteases

In addition to bacterial stimulation of the host’s native sheddases, proteases encoded and secreted by pathogens have also been described to catalyze E-cadherin fragmentation, independent of endogenous enzymes.

#### 3.2.1 High-temperature requirement A (HtrA)

Full-length 125 kDa E-cadherin was identified as a substrate to the serine protease and periplasmic chaperone HtrA, a caseinolytic active enzyme secreted by *H. pylori*. The HtrA-mediated cleavage of the extracellular 90 kDa amino-terminal domain (NTF) of E-cadherin results in the release of CTF1 that, upon further processing, yields a soluble 33 kDa CTF2 fragment (Hoy et al., 2010). A 29 kDa E-cad/CTF3 fragment can be produced by caspase-3 cleavage in *H. pylori*-induced apoptosis of gastric epithelial cells (Yang et al., 2017). HtrA was reported to cleave at the linker regions between the EC domains, with the signature cleavage sites potentially being masked under calcium-binding homophilic homotypic interactions (cis and trans) (Schmidt et al., 2016b, a). HtrA was further characterized as a highly conserved virulence factor among bacterial species, with HtrA-mediated E-cadherin truncation potentially comprising a prominent pathogenic mechanism for Gram-negative gastrointestinal pathogens, including *H. pylori*, *Campylobacter jejuni*, enteropathogenic *Escherichia coli* (*EPEC*), *Shigella flexneri*, *Salmonella enterica subsp. Enterica* (*S. Typhimurium*), *Yersinia enterocolitica*, and *Proteus mirabilis* (Hoy et al., 2012; Abfalter et al., 2016). Of note, HtrA-mediated E-cadherin cleavage properties are limited to DegP and DegQ homologs expressed by Gram-negative pathogens, which harbor different HtrAs combinations (Abfalter et al., 2016). The Hoy group showed that HtrA is expressed mainly as active multimers in *H. pylori* and *C. jejuni* -as opposed to monomers in *EPEC* and *S. flexneri*- allowing the pathogens to efficiently and rapidly transverse polarized MKN-28 monolayers via the paracellular route (Hoy et al., 2012). In *H. pylori* infection, HtrA-mediated E-cadherin shedding on the surface of highly polarized gastric epithelial cells, permits CagA injection and tyrosine phosphorylation in the cytosol of non-transformed healthy cells (Canadas-Ortega et al., 2024). In the case of *C. jejuni*, the transmigration does not confer any drastic reduction in transepithelial electrical resistance (TEER), suggesting that HtrA-directed cell-cell junction opening is executed in a strictly controlled, spatiotemporally restricted manner that enables pathogens to seamlessly cross the intercellular space, whereas this translocation capacity is severely defected in ΔHtrA mutants compared to wild-type bacteria (Boehm et al., 2012). *C. jejuni* outer membrane vesicles (OMVs) with serine protease activity targeting intestinal epithelial E-cadherin and occludin are thought to deploy HtrA to exercise their cleaving effects (Elmi et al., 2016). Yet, the group of Sharafutdinov and colleagues showed by electron and confocal immunofluorescence microscopy that it is not the soluble purified protease nor the protease in HtrA-containing OMVs, but the *C. jejuni* surface-bound HtrA that disrupts epithelial cell-cell junctions (Sharafutdinov et al., 2024). Moreover, HtrA-expressing *C. jejuni* was shown to facilitate co-translocation of commensal microbiota with otherwise weak transmigratory capabilities, such as non-pathogenic *E. coli* and *Lactococcus lactis*, which may represent a central mechanism that underpins the pathogenesis of inflammatory bowel disease (IBD) (Sharafutdinov et al., 2022). Additionally, HtrA induction as a proteolytic tool that manipulates host cell machinery has been reported in chlamydial infection (Wu et al., 2011) and in *Listeria monocytogenes* (Radhakrishnan et al., 2021), while it also plays a role in stress resistance and pathogenicity of *Bacillus anthracis* (Israeli et al., 2019). However, proof of enhanced E-cadherin degradation was not established in these conditions. In *Coxiella burnetii* infection, secretion of functional cbHtrA was pinpointed as another plausible mechanistic explanation behind the elevated sE-cad levels found in sera of patients with Q fever (Mezouar et al., 2019; Osman et al., 2023). Indeed, recombinant cbHtrA-treated and *C. burnetii*-infected BeWo cells released markedly higher sE-cad compared to unstimulated cells, while cbHtrA-exposed macrophages skewed toward M2-polarized interleukin signature which additionally downregulated E-cadherin expression (Osman et al., 2023). *Borrelia burgdorferi*, the causative agent of Lyme disease, is also endowed with HtrA-mediated cleaving capacity *in vitro*, allowing host extracellular matrix (ECM) protein and E-cadherin degradation, which is consistent with spirochaetal dissemination findings (Russell et al., 2013). Lastly, E-cadherin ectodomain shedding by HtrA/DegQ virulence factor has lately been described in porcine respiratory pathogens such as *Glaesserella* (*Haemophilus*) *parasuis* and *Actinobacillus pleuropneumoniae* (Cao et al., 2021; Zhang et al., 2022). Studies have shown that bacterial paracellular transmigration was significantly higher in E-cadherin knock-out, as opposed to the effects of HtrA depletion (Cao et al., 2021).

#### 3.2.2 BFT or fragilysin (FRA)

The group of Wu et al. proved that enterotoxigenic *Bacteroides fragilis* leverages a zinc-dependent metalloprotease toxin termed BFT or fragilysin, that shares homology with eukaryotic MMPs, in order to manifest its virulence through BFT-initiated step-wise cleavage of E-cadherin; extracellular ectodomain shedding (80 kDa) and subsequent proteolytic processing with intracellular fragmentation (i.e., 33 kDa, by presenilin-1/γ-secretase) (Wu et al., 2007). Loss of full-length E-cadherin forces dispersion of E-cadherin-bound β-catenin pool and cytoplasmic localization within 1-3 hours. Upon nuclear translocation (3-24 hours), it activates proliferative signaling via TCF pathway activation and c-myc transcription (Wu et al., 2003). Biologically active BFT, capable of E-cadherin degradation, has been found in OMVs as a bacterial secretory delivery system (Zakharzhevskaya et al., 2017). Fragilysin-catalyzed shedding of intestinal epithelial E-cadherin *in vivo* has been reported to be implicated in murine colitis onset and early IL-8 secretion (Rhee et al., 2009; Lee et al., 2022). Of note, IL-8 induction due to BTF-mediated E-cadherin cleavage is β-catenin-dependent and requires NF-κB signal activation (Lee et al., 2022). MMP-2 was found to be encoded by the same *B. fragilis* pathogenicity island, but E-cadherin was not recognized as a cleavage substrate (Shiryaev et al., 2014). BFT in anaerobic bacteremia and sepsis has a similar functional role to ADAM10 in *S. aureus* sepsis. A clostripain-like *B. fragilis* protease named fragipain is involved in endogenous BTF activation and secretome generation and can directly or indirectly promote E-cadherin-targeted proteolytic activity (Choi et al., 2016; Pierce et al., 2021).

#### 3.2.3 Gelatinase (GelE)

Other microbial metalloproteases impairing full-length E-cadherin have been documented, including a GelE produced by commensal *Enterococcus faecalis* strains; GelE was shown to trigger loss of extracellular E-cadherin and barrier breakage, contributing to the development of experimental colitis in *E. faecalis* mono-associated IL-10^−/−^ mice, irrespective of antigen-specific activation of colitogenic CD4+ T cells (Steck et al., 2011). Ex vivo epithelial permeability induction by purified GelE appears to require PAR2 activation, while human fecal supernatants from ulcerative colitis (UC) patients can enhance colonic epithelial permeability in wild-type mice, while the effects were lower in PAR2^−/−^ mice (Maharshak et al., 2015).

#### 3.2.4 Gingipains

*Porphyromonas gingivalis*, an established pathogen in adult periodontal disease, is known to secrete three cysteine proteases known as gingipains (HRgpA, RgpB, and Kgp). Gingipains are believed to account for the breakdown of E-cadherin by *P. gingivalis*, with Kgp being the major degradative effector (Katz et al., 2002). A plethora of other host proteins’ processing has been ascribed to gingipains, including proMMP-9 (Inaba et al., 2014; Hočevar et al., 2018), while β-catenin can also undergo proteolytic activation attributed to gingipains, in noncanonical (Wnt-independent) fashion (Zhou et al., 2015). In peri-implant disease (i.e., peri-implant mucositis and peri-implantitis), gingipains can interfere with sulcular epithelium attachment to titanium–zirconium alloy surfaces through their cleaving ability (Eick et al., 2019). In the intestinal epithelium, gingipains are thought to be employed in murine colitis exacerbated by orally administered *P. gingivalis* (Tsuzuno et al., 2021).

#### 3.2.5 Miscellaneous bacterial proteases

Other putative microbial cysteine proteases with E-cadherin-cleaving activity have been documented; for instance, *Clostridium perfringens* culture supernatant induced *in vitro* degradation of recombinant E-cadherin -albeit no host protease activation-, while cysteine protease inhibitors completely extinguished the proteolytic effects (Pruteanu and Shanahan, 2013).

An extracellular serine protease of *Mycobacterium tuberculosis* named Rv2569c was recently shown to cleave E-cadherin; M. tuberculosis Rv2569c allowed the bacteria to translocate through the respiratory epithelial barrier *in vivo* and confer pathological damage to murine pulmonary tissues, promoting colonization and systemic dissemination (Zang et al., 2024).

*Leptospira interrogans*, etiological agent of leptospirosis, one of the most significant zoonoses globally, is known to displace E-cadherin from the membrane and drive cytoskeletal rearrangement and AJ disassembly by hijacking the host cells’ ubiquitinin-proteasomal system (UPS) and/or lysosomal degradation pathways. Tokumon and co-workers found that *L. interrogans* specifically triggers E-cadherin endocytosis by mislocalization and degradation of the p120ctn sub-family proteins (p0071 and p120ctn) that interact with the juxtamembrane domain of E-cadherin, through induction of an unidentified protease inhibited by Z-VAD-FMK (Tokumon et al., 2023). The UPS hijacking could also be involved in the degradation of other modulators of cell-cell junctions and cytoskeletal dynamics such as Rho GTPases including Rac1, Cdc42, and RhoA proteins (Tokumon et al., 2023).

Interestingly, a study by Haderer and colleagues investigating the bacterial-to-cell effects in spontaneous bacterial peritonitis (SBP) found that stimulation with *E. coli* and *P. mirabilis* led to the cleavage of E-cadherin through a novel bacterial protease activity. In contrast, intestinal bacteria induced the downregulation of the TJ protein occludin via enhancing endogenous proteasomal degradation in colonic epithelial cells (Haderer et al., 2022).

### 3.3 Transcriptional regulation of E-cadherin

Bacterial pathogens can seemingly affect E-cadherin expression on a transcriptional level as well as subvert epigenetic alterations that lead to junctional disturbances. *P. gingivalis*-lipopolysaccharide (LPS) substantially reduced E-cadherin protein expression in epi-4 cells compared to no *P. gingivalis*-LPS challenge (Abe-Yutori et al., 2017). This expression pattern has been demonstrated in chronic periodontitis subjects, showing a statistically significant decrease in E-cadherin levels compared to healthy individuals, which inversely correlated with K19 increase (Nagarakanti et al., 2007). Semiquantitative immunohistochemical analysis of tissue samples detected a statistically significant reduction in staining intensity from the external oral epithelium, through the gingival sulcus, to the junctional epithelium of clinically healthy gingiva, with the most marked decrease seen in the pathological lining of the pocket epithelium (Ye et al., 2000). In murine gingivitis epithelia, noticeably decreased E-cadherin expression was observed under the inflamed condition on a protein and mRNA level. This was inversely associated with induction of pyroptosis, namely programmed cell death triggered by caspase-1 activation, where caspase-1 and E-cadherin were inversely correlated (Li et al., 2021).

*Clostridium perfringens* beta2 (CPB2) toxin was shown to confer intestinal epithelial barrier injury in porcine IPEC-J2 cells treated with 20 μg/mL rCPB2 by considerably restricting claudin-1 and E-cadherin mRNA and protein expression levels (Gao et al., 2020). In a transcriptomic analysis of human trophoblast cells (BeWo), many junctional protein genes were recognized as differentially expressed in response to *E. faecalis* infection, including E-cadherin, which was found significantly downregulated (Tan et al., 2018). E-cadherin transcripts were measured to be progressively inactivated over time in *Shigella dysenteriae*-infected HT29 cells, with ensuing β-catenin cytoplasmic translocation (Raja et al., 2012).

CDH1 promoter hypermethylation of CpG islands is one of the most common epigenetic patterns that transcriptionally suppress E-cadherin expression. This epigenetic modification is widely considered to have a greater frequency in *H. pylori* chronic gastritis and constitutes an established early event in gastric carcinogenesis (Chan, 2003; Kang et al., 2003; Liu et al., 2005). In a study, methylation density in gastric body and antral mucosae obtained from *H. pylori*-positive gastritis patients was approximately 10-fold higher compared to *H. pylori*-negative patients. The study showed that host inflammatory cytokines and growth factors -including TNF-α, MG132 (ROS), and EGF in response to the infection mediate aberrant E-cadherin methylation and DNA methyltransferase (DNMT) activity *in vitro* (Miyazaki et al., 2007). IL-1β-stimulated NF-κB cascade activation and DNMT induction via NO production is another compelling transcriptional system engaged in *H. pylori*-associated hypermethylation status, which conceivably links chronic gastric inflammation and carcinogenesis (Huang et al., 2012). Successful *H. pylori* eradication therapy notably eliminates methylation effects and results in reversal of prior silencing (Chan, 2006; Leung et al., 2006; Miyazaki et al., 2007), potentially reinstating E-cadherin expression-dependent barrier function. Interestingly, the opportunistic pathogen *Acinetobacter baumannii* was also found capable of hindering E-cadherin expression through promoter CpG methylation following its nuclear trafficking (Moon et al., 2012). In the pathophysiological course of *Chlamydia trachomatis* infection, EMT induction also seems to entail methylation increment in the E-cadherin promoter, while upregulation of other mesenchymal markers was not proven to stem from significant epigenetic alterations (Rajić et al., 2017).

### 3.4 Interactions involving the extracellular domain of E-cadherin

Given that the extracellular part of E-cadherin engages in homotypic and heterotypic interactions to achieve cell aggregation and control cell behavior, bacteria can seize the molecule’s ectodomain as a heterophilic receptor for adherence and uptake by host cells. *L. monocytogenes*, a food-borne pathogen able of prototypic intracytosolic invasion in non-phagocytic cells, can employ a well-described invasion protein named internalin (lnlA) to interact with the N-terminal EC1 domain via a leucine-rich repeat (LRR) of the bacterial ligand, securing attachment and internalization at the site of the bacterial-epithelial interface (Mengaud et al., 1996; Schubert et al., 2002). Upon specific calcium-requiring anchoring to E-cadherin, *L. monocytogenes* can initiate lnlA-based and locally constrained entry into the epithelial cells at the sites of bacterial contact without inducing dramatic morphological changes. This type of bacterial ligand-promoted endocytosis more closely resembles the “zipper mechanism” of *Yersinia* entry but is distinct from *Salmonella* “trigger” invasion mechanism (Mengaud et al., 1996). Bonazzi et al. showed that lnlA attachment induces sequential E-cadherin post-translational modifications, which are prerequisites for the recruitment of the different components of endocytosis machinery at the bacterial entry site. In this regard, induced Src-mediated phosphorylation and ubiquitination by ubiquitin-ligase Hakai at the juxtamembrane E-cadherin domain were required for caveolin-dependent E-cadherin clustering and clathrin-mediated internalization (Bonazzi et al., 2008). In fetoplacental listeriosis, *L. monocytogenes* crosses the maternofetal or trophoblastic barrier via heterotypic interaction between accessible syncytiotrophoblast E-cadherin with lnlA, as recapitulated *ex vivo* in human placental extracts (Lecuit et al., 2004). In the intestinal villi, where E-cadherin is naturally basolateral and secluded from the lumen, *L. monocytogenes* was shown to exploit transient defects of epithelial polarity and junctional remodeling spots to facilitate penetration. Indeed, multicellular junctions formation in cell extrusion zones of villus tip can function as entry points and enable the pathogen to efficiently reach the apically exposed E-cadherin prior to its dynamin-dependent removal from the cell surface (Pentecost et al., 2010). Apart from extruding apoptotic cells on villi tips or cells located within intestinal epithelial folds, reorganization of apical junctional complexes around goblet cells, which is affected by physical tensions associated with mucus-expelling dynamics, can similarly make E-cadherin luminally accessible. This allows lnlA-initiated rapid transcytosis across intestinal villi vertical axis with ultimate bacterial release from the basal pole of enterocytes into the lamina propria (Nikitas et al., 2011). Of note, even though InlA binding to E-cadherin is indispensable and adequate for *Listeria* attachment, modulation by another internalin (InlB) expedites invasion through the displaced junctions and synergistically promotes endocytosis through activation of c-Met signaling (Pentecost et al., 2010). Ultimately, the strength of lnlA-E-cadherin interaction per se may not directly correlate with the invasive capacity, conceivably reflecting lnlA’s non-exclusive role in determining *L. monocytogenes* virulence (Dellafiora et al., 2020). Intriguingly, invasion can also involve other host cell-dependent mechanisms such as cell membrane perforation to hijack the endocytic machinery by use of pore-forming exotoxin listeriolysin O; extracellular Ca^2+^ influx and Rac1 activation-dependent downstream signaling lead to actin cytoskeleton *de novo* assembly mandated for *Listeria*’s internalization (Lam et al., 2018).

Pneumococcal surface adhesin A (PsaA) of *Streptococcus pneumoniae* has been identified as another heterophilic ligand of E-cadherin during the initial stage of bacterial colonization in the nasopharyngeal (NP) epithelium. PsaA binding was found to be calcium-dependent and, unlike lnlA that is specific to human E-cadherin, both human and mouse E-cadherin were able to inhibit adherence of PsaA-coated fluospheres to NP cells (Anderton et al., 2007).

Login et al. demonstrated that EPEC microcolonies also recruit E-cadherin at intercellular junctions of polarized and nonpolarized cells. However, only after the initial establishment of interaction between bacterial intimin and the Tir receptor on the host membrane, is E-cadherin able to bind to the Tir-intimin complex. Nonetheless, E-cadherin still influences EPEC attachment as the absence of the extracellular domain of E-cadherin significantly reduced EPEC adhesiveness (Login et al., 2018).

Another adhesin, FadA, was described to bind to the EC5 domain of E-cadherin, promoting attachment and invasion in colorectal cancer (CRC) and non-CRC cells under *Fusobacterium nucleatum* infection. In CRC cells, interaction of E-cadherin with FadA was shown to induce downstream β-catenin signaling. Specifically, E-cadherin phosphorylation, internalization of the complex, cytoplasmic translocation of β-catenin, and transcriptional activation of Wnt/β-catenin target genes were shown to be induced (Rubinstein et al., 2013). *F. nucleatum* may thus promote the malignant phenotype of CRC by enhancing tumor growth, inflammatory responses, and EMT through interaction with E-cadherin. However, *F. nucleatum* only increased the inflammatory responses when β-catenin expression was knocked down in normal colonic cells, whereas no changes were observed when E-cadherin expression was knocked down (Ma et al., 2018).

Bacterial toxins have also been shown to associate with the E-cadherin receptor, disrupting the intercellular epithelial continuity to allow their subsequent uptake. Hemagglutinin (HA) constitutes a nontoxic accessory component of the botulinum neurotoxin complex, produced by *Clostridium botulinum* and known to cause flaccid paralysis in animals and humans. HA was found to bind to E-cadherin on EC1-EC2 residues in a species-specific manner, disrupting its function by sterically blocking E-cadherin trans-dimerization (Sugawara et al., 2010; Lee et al., 2014).

*H. pylori* virulence factor CagA can also interact physically with E-cadherin, functionally impairing E-cadherin/β-catenin complex assembly in gastric epithelial cells independently of CagA tyrosine phosphorylation status. The resultant β-catenin cytosolic and nuclear accumulation can transactivate β-catenin-regulated signaling, including intestinal-specific transdifferentiation genes, implicated in metaplasia and gastric carcinogenesis (Murata-Kamiya et al., 2007). Oliveira and colleagues later suggested that CagA interacts with E-cadherin and p120ctn in a c-Met-dependent manner, promoting multiprotein formation between CagA, c-Met, E-cadherin, and p120ctn. This interestingly inhibits c-Met and p120ctn phosphorylation and restrains the invasive phenotype induced by *H. pylori* (Oliveira et al., 2009).

Interestingly, E-cadherin was found to serve as an adherence receptor for *C. difficile* spores onto intestinal epithelial cells (IECs). Castro-Córdova et al. observed that E-cadherin was able to bind to the hairlike projections of the spores, and that the E-cadherin-specific interaction with IECs was toxin-mediated, requiring TcdA and TcdB to open the AJs and render E-cadherin accessible for tethering (Castro-Córdova et al., 2023).

Cadherins have been previously described as able receptors for *Leptospira* (Evangelista et al., 2014). Pereira et al. identified E-cadherin as a binding receptor for protein Lsa16 of *L. interrogans* (Pereira et al., 2017). Kochi and co-workers reported that two putative leptospiral surface-exposed lipoproteins LIC11711 and LIC12587, conserved among pathogenic strains of *L. interrogans*, show binding affinity to E-cadherin in a dose-dependent interaction that allows initial bacterial attachment to host epithelial cells (Kochi et al., 2019). Potential host cell membrane injury and E-cadherin expression changes following leptospirotic attachment have been previously described. Cell membrane insult as the primary cellular lesion of leptospirosis was corroborated immunohistochemically, with E-cadherin expression irregularities in leptospirotic patients and loss of membrane E-cadherin in hepatocytes, associated with liver-plate disarray (De Brito et al., 2006). Strikingly, this E-cadherin downregulation might be attributed to feedback inhibition mechanisms that eventually decrease *Leptospira* colonization. It has been described that LRR proteins expressed by the pathogenic *Leptospira* species can interact with E-cadherin on the host cell surface, inducing E-cadherin/β-catenin and NF-κB signaling cross-talk that can ultimately dictate the fate of E-cadherin and regulate *Leptospira* adhesion and invasion in kidney (Hsu et al., 2021).

## 4 E-cadherin in inflammation and disease pathogenesis

**Figure 4:**
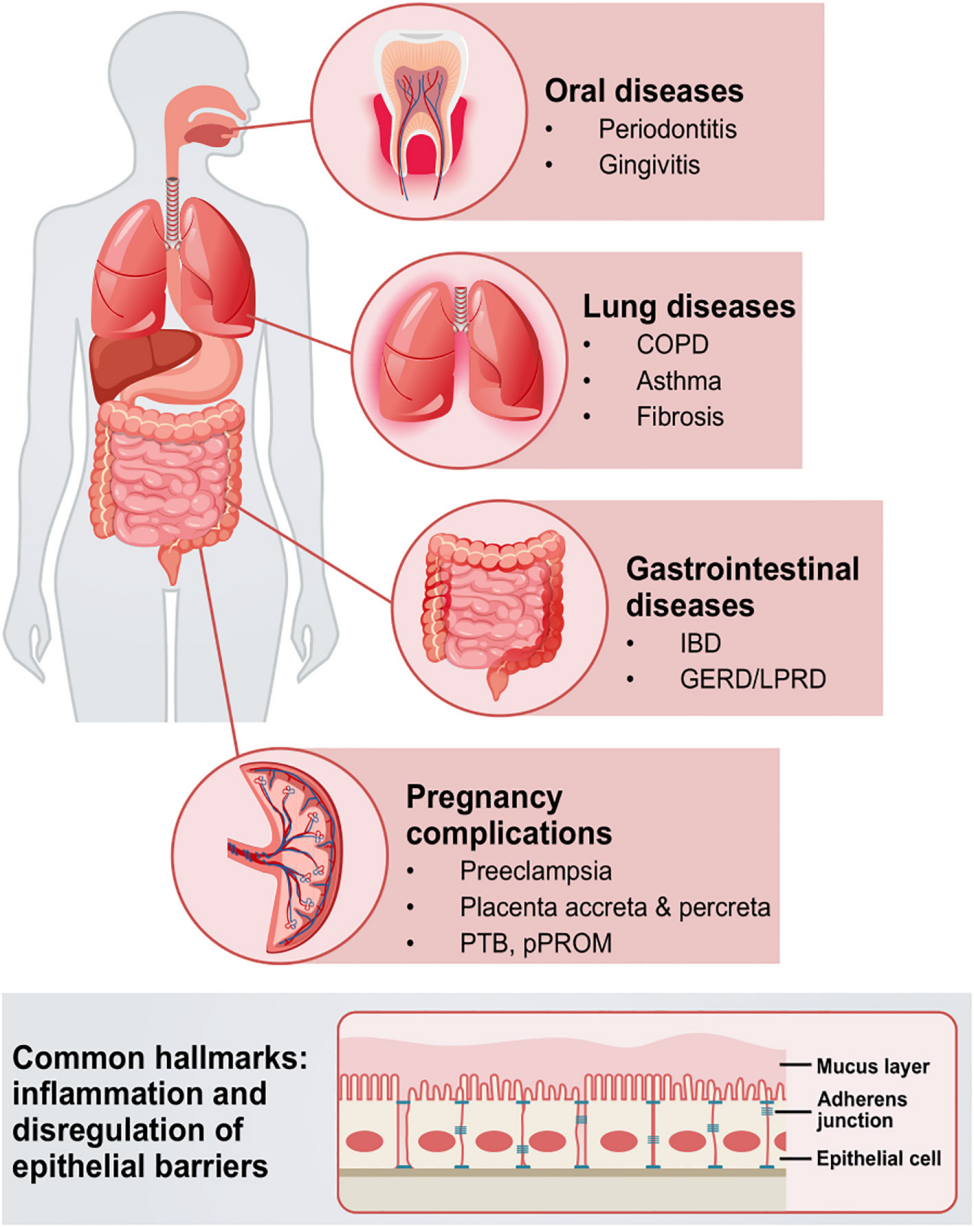
Schematic overview of the systematic implications of epithelial barrier disruption via E-cadherin in different diseases. Pathologies in oral and gastrointestinal mucosae, as well as placenta, lungs, and other tissues and organs, are complex, intertwined entities, that can manifest with barrier dysfunction, inflammation, and/or EMT hallmarks. Such pathologies can engage microbiota as well as underlying immunological components, that collectively drive and aggravate barrier dysfunction. COPD; chronic obstructive pulmonary disease; IBD, inflammatory bowel disease; GERD, gastroesophageal reflux disease; LPRD, laryngophangeal reflux disease; PTB, preterm birth; pPROM, preterm pre-labor rupture of the membranes; EMT, epithelial-to-mesenchymal transition.

Ongoing research is increasingly focused on elucidating the role of E-cadherin in initiation and perpetuation of inflammatory processes and other diseases, in a multitude of epithelial tissues and organs, given its ubiquitous presence. E-cadherin as a peculiar immunomodulatory player in inflammation remains largely underexplored, and its regulator properties that dictate the fine balance between immunity and tolerance remain obscure. Herein, we report the role of E-cadherin, which mediates the functional coupling between epithelial cells, and its effects on barrier dysfunction in various tissues and organs, including the lungs, oral mucosa, the intestine, and the placenta (**Figure 4**).

### 4.1 E-cadherin in lung diseases

A range of lung diseases, including idiopathic pulmonary fibrosis, COPD, and asthma have been associated with loss of E-cadherin function and elevated sE-cad levels (Yuksel et al., 2021; Mottais et al., 2023). Although E-cadherin loss and/or proteolytic processing are observed in inflammatory conditions, it remains unclear whether these changes are a primary cause of disease pathophysiology or simply a secondary response.

*In vitro* and *in vivo* lung injury studies have demonstrated that MMP-7 mediates the cleavage of extracellular E-cadherin, promoting epithelial repair and facilitating cell migration through the redistribution of E-cadherin-based adhesions in wounded epithelium (McGuire et al., 2003). Interestingly, E-cadherin interaction with the αEβ7-integrin receptor or CD103, both of which are expressed on pulmonary anti-fibrotic DCs, is regulated by MMP-7. This interaction promotes the resolution of acute neutrophilic inflammation and induces an anti-inflammatory cytokine profile, thereby balancing epithelial repair with immune activation (Manicone et al., 2009). Interestingly, sE-cad levels were significantly elevated in the BAL fluids and serum of mice with bleomycin-induced pulmonary fibrosis. sE-cad promotes EMT in the alveolar epithelium and abnormal fibroblast migration. Blocking sE-cad effectively reduced myofibroblast accumulation and collagen deposition in the lungs following bleomycin exposure. Additionally, transforming growth factor-β1 (TGF-β1) was found to stimulate the shedding of sE-cad from A549 cells and promote EMT, with these effects being reversed upon sE-cad inhibition (Huang et al., 2024).

Studies by Ghosh et al. have shown loss of E-cadherin in the lung epithelium of patients with COPD. Ghosh et al. reported that knockout of E-cadherin in alveolar epithelial type II but not type I cells in adult mouse models results in airspace enlargement. Furthermore, the knockout of E-cadherin in airway ciliated cells, but not club cells, increases airway hyperreactivity (Ghosh et al., 2022). Additionally, cigarette smoke-induced epithelial injury has previously been linked to E-cadherin-related barrier dysfunction (Nishida et al., 2017; Ghosh et al., 2020). As anticipated, significantly higher levels of sE-cad were found in the plasma of COPD patients and symptomatic smokers compared to healthy smokers and nonsmokers. Moreover, both plasma and epithelial lining fluid (ELF) sE-cad levels were positively correlated with the severity of airway limitation, with ELF sE-cad levels showing a particularly strong correlation with MMP-7 levels (Shirahata et al., 2018).

In the context of asthma development, common environmental factors such as air pollutants are known to impair the airway epithelial barrier by reducing E-cadherin expression. Exposure to sub-toxic levels of soluble PM2.5, diesel exhaust, and other reactive oxygen species (ROS)-generating pollutants has been shown to decrease E-cadherin levels. This reduction in E-cadherin contributes to airway barrier dysfunction, which can increase susceptibility to bacterial infections. The silencing of the E-cadherin gene due to air pollutants may be mediated by dysregulated non-coding RNAs, which are overexpressed in asthma and COPD patients (Aghapour et al., 2022). A study by Michaudel et al. demonstrated that ozone-induced respiratory barrier injury—characterized by protein leak, epithelial cell desquamation, and the recruitment of neutrophils and alveolar macrophages—precedes myeloid cell-driven lung inflammation, bypassing the protective effects of the IL-33/ST2 axis. Acute ozone exposure disrupts IL-33-dependent homeostasis, leading to decreased epithelial E-cadherin expression and increased inflammatory cell infiltration in the absence of ST2 and IL-33. Additionally, the deposition of air pollutants leads to E-cadherin depletion via an HMGB1-mediated mechanism, contributing to abnormal alveolar cell turnover in emphysema (Michaudel et al., 2018). Also it has been reported that loss of E-cadherin upon pollutant exposure triggers cell senescence, chronic disruption of alveolar differentiation, and apoptosis through downstream effectors of the Hippo pathway, such as YAP/TAZ (Chang et al., 2022). Furthermore, recent studies have also linked the upregulation of FcϵRI, monomeric IgE, and IgE/FcϵRI engagement with decreased junctional distribution of E-cadherin in severe asthma. The crosstalk between FcϵRI and EGFR was found to be associated with E-cadherin loss, triggering IL-33 synthesis and release upon IgE-induced EGFR activation (Weng et al., 2023). Heijink and colleagues observed that EGFR phosphorylation and activation following E-cadherin silencing drives EGFR-dependent recruitment of Th2 cells in allergic asthma, through the induction of TARC/CCL2, a Th2-attracting molecule (Heijink et al., 2007). Another mechanism by which environmental factors disrupt the epithelial barrier involves proteolytically active allergens that cleave E-cadherin, either directly through proteolytic activity or indirectly by triggering pattern-recognition receptors (PRRs). Protease allergens activate innate immune receptors such as protease-activated receptors (PARs) and stimulate non-IgE-mediated reactions, leading to the release of mediators (Yuksel et al., 2021). For example, mite allergens induce proteolysis of ZO-1, occludin, and other TJ proteins, while proteases released by pollen disrupt E-cadherin and TJ proteins like occludin and claudin-1. Moreover, proteases found in mite, fungi, and cockroach extracts activate PAR1/2, which subsequently leads to the degradation of E-cadherin (Yuksel et al., 2021). Finally, higher sE-cad levels are associated with more severe asthma, correlating with sputum HMGB1 level and glucocorticoid dosage required for daily management. In addition to that, sputum sE-cad levels reflect asthma severity and inversely correlate with decreases in FEV1 (Masuyama et al., 2003). Upon allergen exposure, significant increases in sE-cad levels were observed in the BAL fluids of mice. It is believed that sE-cad contributes to airway inflammation in severe asthma through ERK signaling, which upregulates VEGF and IL-6, and promotes the infiltration of neutrophils and eosinophils into the airways (Tang et al., 2024).

### 4.2 E-cadherin in oral diseases

Gingivitis and periodontitis are oral diseases characterized by dysbiosis, periodontium destruction, and aberrant immune responses of the host. In chronic inflammatory conditions, (i.e., periodontitis), E-cadherin expression in epithelium is significantly downregulated during pocket formation (Nagarakanti et al., 2007; Saliem et al., 2023). Notably, elevated sE-cad salivary levels were shown to positively correlate with periodontitis severity (Kazem et al., 2023). Notably, gingival crevicular fluid (GCF) E-cadherin significantly increased in gingivitis and periodontitis cases as compared to controls (Hussein et al., 2024b). E-cadherin levels in GCF has been shown to be a good predictor for nonsurgical periodontal therapy outcomes in periodontitis patients (Hussein et al., 2024a).

Several mechanisms are involved in the regulation of E-cadherin in periodontitis. Specifically, a study by Hiyoshi et al. has shown that NE disrupts the gingival epithelial barrier by degrading E-cadherin, allowing periodontal pathogens to penetrate the periodontal tissues (Hiyoshi et al., 2022). Also, in the pathological epithelial lining of periodontal pockets, the reduction of E-cadherin has been linked to the EMT phenotype (Saliem et al., 2022, 2023; Kadeh et al., 2023). In an epigenetic study, hypermethylation of CpG islands in the CDH1 gene was detected in 25% of patients with chronic periodontitis, whereas no such hypermethylation was observed in healthy individuals (Loo et al., 2010). In this context, epigenetic silencing has been previously shown to contribute to the inactivation of E-cadherin (Strathdee, 2002). Finally, physiological mechanical stress (e.g., mastication, interstitial pressure, and dental manipulations) compromised E-cadherin intracellular levels and translocation (Lee et al., 2023; Vitkov et al., 2023).

### 4.3 E-cadherin in gastrointestinal mucosal diseases

E-cadherin plays a crucial role in maintaining intestinal epithelial function and regulating the inflammatory immune response. Disruption of E-cadherin-mediated cell-cell adhesion has been linked to increased intestinal permeability, commonly referred to as “leaky gut,” as well as enhanced infiltration of inflammatory cells—two key pathophysiological features of IBD. E-cadherin deficiency is associated with more pronounced colitis and histopathological changes related to tissue repair, as well as increased disease severity (Grill et al., 2015). Reduced E-cadherin expression correlates with the duration and severity of symptoms in IBD patients (Wilcz-Villega et al., 2014). In mucosal samples from patients with chronic active UC, decreased E-cadherin was primarily observed at the lateral membranes of enterocytes, particularly near sites of active PMN transmigration (Kucharzik et al., 2001). Furthermore, Motta et al. (2021) identified elastase 2A (ELA2A), an epithelium-derived elastase distinct from leukocyte-secreted NE, and linked epithelial elastolytic overload in colonic cells of IBD patients to E-cadherin degradation. ELA2A hyperactivity was associated with a pro-inflammatory phenotype, leading to dysregulation of the cytokine profile (e.g., upregulation of IL-8/CXCL8, a neutrophil chemoattractant) and activation of intestinal mucosal immunity (Motta et al., 2021). In addition to the “autocrine” regulation of downstream signaling processes caused by the post-shedding E-cadherin disarrangement, a “paracrine” loop involving the released soluble fragments has also been hinted (Hu et al., 2016). Intriguingly, E-cadherin peptide fragments themselves -found in chronic inflammatory states such as IBD- possess biological properties that contribute to mucosal wound healing (Gordon et al., 2019).

Exacerbated mucosal injury in adulthood may result from neonatal stressor exposure and associated epigenetic changes. Specific CDH1 polymorphisms, such as the CDH1 GTC risk haplotype (a 3-SNP haplotype: rs12597188, rs10431923, and rs9935563), which has an estimated allelic frequency of 21%, have been linked to abnormal E-cadherin trafficking and are significantly associated with an increased susceptibility to Crohn’s disease (Muise et al., 2009). Elevated miRNA expression is a hallmark of inflammation and EMT in IBD and is inversely correlated with CDH1 expression in inflamed mucosa (Guz et al., 2020). In a “dual insult” model of neonatal and adult colonic inflammation, TNF-α-regulated epigenetic activation of miRNA-155 (miR-155) was found to significantly suppress E-cadherin expression for a prolonged period, compared to both single insult and control groups (Kline et al., 2020). A study by Tian has shown that upregulation of miR-155 inhibits post-transcriptional E-cadherin protein synthesis through a RhoA-dependent mechanism (Tian, 2013). Also, miR-21a-5p has been shown to be upregulated in exosomes derived from intraperitoneal macrophages in a DSS-induced enteritis model. A negative correlation was observed between exosomal miR-21a-5p and E-cadherin expression in enterocytes (Lu et al., 2021).

The adhesive properties of E-cadherin on immune cells, such as DCs, are also implicated in colitis development. Ihara et al. (2018) found that E-cadherin was upregulated in a tissue-resident subset of lamina propria CD11c+ DCs in CD11c-Cre TGF-βr2fl/fl mice. E-cadherin-mediated interactions between CD11c+ monocytes and the intestinal epithelium promoted Notch signaling activation. When combined with the abrogated inhibitory effects of TGF-β, this interaction was colitogenic, driving dysbiosis and abnormal epithelial differentiation (Ihara et al., 2018). Additionally, the homing of E-cadherin+CD11+ monocyte-derived DCs to mesenteric lymph nodes in colitic mice may play a key role in T-cell-mediated gut inflammation, with TGF-β appearing to limit this effect (Siddiqui et al., 2010). These DCs can activate naïve T-cells through robust cytokine and chemokine secretion. Adoptive transfer of these cells to immunodeficient hosts led to the expansion of the E-cadherin+ DC population at sites of accumulation and promoted Th17 responses. Notably, this subset exhibited high MHC II expression, along with significantly elevated levels of toll-like receptors and CCR2, compared to E-cadherin (-) DCs, highlighting their heightened sensitivity to microbial triggers and increased inflammatory potential (Siddiqui et al., 2010). Furthermore, E-cadherin was found to engage in inhibitory interactions with KLRG1 on group 2 innate lymphoid cells (ILC2). Upon E-cadherin depletion, this interaction is disrupted, resulting in increased Th2 cytokine levels and excessive ILC2 induction (Lu et al., 2021).

Finally, epithelial barrier dysfunction via E-cadherin proteolysis has been increasingly implicated in the pathogenesis of gastroesophageal reflux disease (GERD) (Jovov et al., 2011; Samuels et al., 2023; Lu et al., 2024). The presence of a 35-kDa intracellular C-terminal fragment and an increase in soluble N-terminal fragments of E-cadherin in sera of GERD patients have been previously reported. This is attributed to ADAM10-mediated cleavage of E-cadherin, which leads to enhanced esophageal epithelial permeability (Jovov et al., 2011). Also, pepsin-pH4 has been shown to cause E-cadherin fragmentation, which is not salvaged by known E-cadherin sheddase inhibitors. Acidified pepsin can cleave full-length E-cadherin (125 kDa), resulting in 38 and 33 kDa C-terminal E-cad/CTF1 and E-cad/CTF2 fragments, respectively, indicative of regulated intramembrane proteolysis (RIP). Furthermore, it can induce ADAM10 maturation and drive transcriptional targets of E-cadherin RIP fragments such as MMPs (Samuels et al., 2023). Aside from GERD, E-cadherin downregulation has also been reported in the pathogenesis of laryngopharyngeal reflux disease (LPRD), with increased levels of MMP-7-mediated degradation being observed in LPRD biopsies (Reichel et al., 2008; Im et al., 2022).

### 4.4 E-cadherin in pregnancy complications

Spontaneous preterm birth (PTB) and preterm pre-labor rupture of the membranes (pPROM) are major pregnancy complications where E-cadherin alterations have been implicated as part of the EMT process (López-Novoa and Nieto, 2009; Sisto et al., 2021; Menon, 2022). Interestingly, preterm labor is triggered by EMT-associated inflammation and immune imbalances at the fetomaternal interface (Menon et al., 2020). Human amnion cells can undergo non-canonical EMT, including the downregulation of E-cadherin, in response to inflammatory mediators such as TNF-α. This process predisposes the fetal membranes to weakening, increasing the risk of preterm birth (De Castro Silva et al., 2020).

Pre-eclampsia (PE) is also a common pregnancy complication involving an inflammatory phenotype and immune perturbations at the fetoplacental unit (Cornelius, 2018; Michalczyk et al., 2020). E-cadherin shedding, regulation, and transport play crucial roles in trophoblast differentiation, fusion, and physiological placental formation (Shih et al., 2002; Aghababaei et al., 2015; Iwahashi et al., 2018). However, preeclamptic extravillous trophoblasts showed a decrease in their E-cadherin expression indicating the significance of E-cadherin in trophoblast function (Blechschmidt et al., 2007). Mechanistic studies in early-onset PE placental tissues showed that E-cadherin expression is associated with the downregulation of ribosomal protein L39 and the loss of its suppressive control (Jie et al., 2021). Circular RNAs and miRNAs have also been implicated in modulating E-cadherin expression, contributing to the molecular events underlying PE pathogenesis (Zhu et al., 2020).

Although E-cadherin expression typically declines during progressing gestation, in pregnancies complicated by PE, placental E-cadherin levels significantly increase at the protein level. This may reflect abnormal cytotrophoblast proliferation relative to syncytiotrophoblasts, indicating an imbalance in the trophoblastic proliferative unit (Brown et al., 2005). This aligns with a study by Benian et al., which displayed that elevated E-cadherin levels, as well as IL-10 and TGF-β1, were significantly higher, were significantly higher in plasma and placentae of PE patients (Benian, 2002). Immunohistochemical discontinuity of E-cadherin expression in the syncytiotrophoblastic basal membrane can constitute a marker of impaired placental barrier integrity, and by extension pregnancy-induced hypertension or PE (Pęksa et al., 2022). Despite that, E-cadherin upregulation in the syncytiotrophoblast of preeclamptic placentae has not been considered a disease severity marker (Li et al., 2014).

In placenta accreta and percreta, trophoblastic E-cadherin is significantly reduced (Duzyj et al., 2015; Incebiyik et al., 2016). Similarly, the reduction in E-cadherin expression of placental villi has been reported in gestational trophoblastic diseases (Li et al., 2003; Xue et al., 2003). Loss of E-cadherin, induced by Snail upregulation under hypoxic conditions, can activate α5-integrin signaling and promote extravillous trophoblast invasiveness (Arimoto-Ishida et al., 2009).

### 4.5 E-cadherin in other diseases

E-cadherin plays an important role in pancreatitis and autodigestive inflammatory diseases. Specifically, cathepsin C (CTSC) has been reported as an activator of NE, which degrades E-cadherin. Notably, in models with CTSC deletion, E-cadherin cleavage—though not neutrophil motility—was reduced, resulting in milder disease (John et al., 2019). Importantly, E-cadherin breakdown can be entirely mediated by NE, without the need for the proteolytic activity of native pancreatic enzymes (Mayerle et al., 2005).

In atopic dermatitis, E-cadherin has been identified as a proteolytic substrate of granzyme B, a serine protease that, along with perforin, is known to mediate lymphocyte-induced apoptosis (Turner et al., 2021). This suggests that E-cadherin functions as a “double-faced” molecule, playing roles in both adhesion and signaling. Its degradation, particularly during neutrophil transmigration mediated by NE, not only causes epithelial injury but also promotes the proliferation of surviving epithelial cells to facilitate repair or potentially drive pathological remodeling. The shedding of E-cadherin’s ectodomain ultimately supports re-epithelialization by promoting β-catenin signaling and its translocation to the nucleus, potentially upregulating canonical Wnt signaling to mitigate collateral epithelial damage (Zemans et al., 2011).

Several proteolytic cascades are involved in E-cadherin degradation and the disassembly of AJs, including a variety of enzymes such as zinc-dependent MMPs, ADAMs, cathepsins, kallikrein-7, plasmin, and calpain, all of which catalyze the proteolytic cleavage of E-cadherin (Rios-Doria et al., 2003; Grabowska, 2012). In eczematous dermatitis, soluble stimuli like LPS, proinflammatory cytokines, and TGF-β significantly increase ADAM10-dependent E-cadherin shedding, impairing keratinocyte cohesion and contributing to the disease’s molecular pathology through the activation of MAPK signaling, which regulates sE-cad release (Maretzky et al., 2008). Of note, enhanced metalloprotease-catalyzed production of sE-cad has also been linked to EGFR activation (Zuo et al., 2011). Furthermore, ADAM15-mediated ectodomain shedding plays a role in stabilizing HER2 and HER3 heterodimerization, leading to receptor activation and proliferative signaling (Najy et al., 2008).

In renal tissue damage and inflammation, E-cadherin is found to be down-regulated upon cisplatin-induced acute renal injury (AKI), whereas E-cadherin levels amelioration is suggested to alleviate the inflammatory effects and rescue from AKI (Gao et al., 2018). E-cadherin overexpression in M2 macrophages (IL-4/IL-13-induced, alternatively activated macrophages) has been shown to attenuate the inflammatory cytokine response to LPS stimulation, indicating a protective, anti-inflammatory role of E-cadherin on immune cells (Van Den Bossche et al., 2015). Conversely, a pro-inflammatory capacity of sE-cad has been identified, contributing to TNF-α production in synovitis via its interaction with lectin receptor LRG1 on T-cells (Lode Melis et al., 2014).

In prostate tissue, intact membrane E-cadherin has been found to be considerably downregulated with age and inflammation (Pascal et al., 2021). In fact, one of the hallmarks of benign prostate hyperplasia, termed “inflammaging” (i.e., chronic slow-progressing inflammation in the aging prostate), was phenotypically enhanced even in E-cadherin deficient mice without complete deletion (CDH1^+/-^ mice), accompanied by increased prostatic macrophage infiltration and bladder overactivity (Pascal et al., 2022).

In posterior capsular opacification, a complication of cataract surgery, proliferation, migration, and EMT/fibrotic characters of residual lens epithelial cells are observed. IL-8 seems to promote EMT by mediating CXCR1/2/NF-κB/p65 signal and subsequent RhoA activation, suppressing the expression of E-cadherin and ZO-1 to facilitate cell migration (Si et al., 2024). Downregulation of junctional proteins, including E-cadherin, claudins and occludin has been reported in other scar epithelia, including idiopathic subglottic stenosis (Berges et al., 2024).

## 5 Therapeutic strategies for barrier restoration/rescue of E-cadherin

Understanding the molecular mechanisms that regulate E-cadherin function is crucial for developing novel therapeutic strategies aimed at preserving epithelial barrier integrity and preventing bacterial infections. Several approaches have been proposed to modulate or restore E-cadherin function, which is essential for maintaining epithelial integrity and preventing disease progression. Various modalities have been explored, including small molecules and compounds that stabilize the E-cadherin-catenin complex (Tafrihi and Nakhaei Sistani, 2017), cadherin and cadherin-mimetic peptides (Li et al., 2019; He et al., 2020), and antibodies that target specific cadherins (Micalizzi et al., 2022). These strategies have shown great potential for treating diseases linked to impaired epithelial barriers and for restoring E-cadherin function. Below, we discuss potential treatments aimed at enhancing E-cadherin expression and improving epithelial barrier function (**Table 3**).

**Table 3:** Summary of various therapeutic strategies aimed at restoring and stabilizing the epithelial barrier and E-cadherin function.

Therapeutic Approaches	Description	References
**Vitamin Supplementation**	**Vitamin D** reinforces E-cadherin junctions by suppressing TNF-α-induced NF-κB signaling, reducing MMP-9 production, regulating EMT, and modulating TGF-β and Wnt/β-catenin pathways. Vitamin D in its **1.25(OH)2D3** form rescues E-cadherin expression and enhances β-catenin binding. **MART-10**, a noncalcemic calcitriol analogue, inhibits MMP-2 and MMP-9 synthesis and blocks EMT by bolstering E-cadherin expression.	(Chiang et al., 2014; Xin et al., 2017; Zhao et al., 2018; Oh et al., 2019; Sari et al., 2020)
**Microbial Metabolites and Commensal Microorganisms**	**HYA** from **Lactobacillus spp.** restores TJ molecules, reduces inflammation, and protects E-cadherin from proteolysis. **Akkermansia muciniphila** reduces P. gingivalis-induced bone destruction and inflammation and enhances junctional marker expression. **Lactobacillus gasseri ATCC33323** safeguards the intestinal barrier, reduces inflammation, and bolsters the expression of E-cadherin and other junctional markers.	(Miyamoto et al., 2015; Yamada et al., 2018; Huck et al., 2020; Qian et al., 2024)
**Degradation Blockade and Protease Inhibitors**	**BB-94** inhibits E-cadherin-degrading MMPs. **GI254023X**, an ADAM10 inhibitor, prevents E-cadherin shedding and β-catenin translocation. **Amprenavir**, an HIV protease inhibitor, rescues the esophageal epithelial barrier from acidified pepsin-mediated disruption.	(Maretzky et al., 2005; Haderer et al., 2022; Blaine-Sauer et al., 2023)
**Antibody-based Modalities**	**E-cadherin monoclonal antibodies** (mAbs) enhance epithelial barrier function and limit IBD progression. **E-cadherin activating mAbs** reduce loss of barrier function and inflammatory progression in IBD.	(Bandyopadhyay et al., 2021)
**Miscellaneous**	**Banxia Xiexin Decoction** inhibits F. nucleatum colonization and E-cadherin/β-catenin signaling in colitis-to-cancer progression. **Chitosan (Q)** modulates E-cadherin-αEβ7 axis, enhances epithelial cell migration and wound healing, and increases E-cadherin expression. Non-viable heat-killed bacteria exposure such as **tyndallized bacteria** significantly enhances E-cadherin levels in bronchial cells and reinforces airway epithelium´s barrier function and repair potential, potentially counteracting EMT. **Ferrostatin-1** inhibits allergen and pollutant-caused ferroptosis and allows E-cadherin recovery *in vitro* and *in vivo*.	(Di Vincenzo et al., 2024; Jiang et al., 2024; Ma et al., 2024; Moine et al., 2024)

Therapeutic approaches and highlighted modalities are indicated in bold.

TNF-α, tumor necrosis factor alpha; NF-κB, nuclear factor kappa B; MMP, matrix metalloproteinase; EMT, epithelial-to-mesenchymal transition; TGF-β, transforming growth factor β1; 19-nor-2a-(3-hydroxypropyl)-1a,25-Dihydroxyvitamin D3; HYA, 10-hydroxy-cis-12-octadecenoic acid; TJ, tight junction; BB-94, batimastat; ADAM, A- disintegrin and metalloproteinase; HIV, human immunodeficiency virus; mAbs, monoclonal antibodies; IBD, inflammatory bowel disease.

### 5.1 Vitamin D supplementation

Interestingly, supplemental vitamin D was recently reported to reinforce E-cadherin-based junctions by suppressing TNF-α-induced NF-κB signaling and consequently downregulating degradative MMP-9 production *in vitro* (Oh et al., 2019). Vitamin D can regress LPS-triggered inflammation in oral keratinocytes by hindering NF-κB activation (Zhao et al., 2018). Vitamin D in its 1.25(OH)_2_D_3_ form is also known to regulate EMT and activity of TGF-β and Wnt/β-catenin signaling pathways, in addition to controlling E-cadherin turnover through modulating expression profiles of effectors on E-cadherin degradation and membranal stabilization, like p120ctn, Kaiso, and NEDD9 (Sari et al., 2020). Promisingly, vitamin D treatment exerts its protective effects *in vivo* by rescuing E-cadherin expression and enhancing binding affinity and membranal sequestration of β-catenin in conjunction with attenuating transcriptional activity and nuclear fraction of the latter (Xin et al., 2017). MART-10, a noncalcemic calcitriol analogue, significantly inhibited MMP-2 and MMP-9 synthesis more potently compared to 1α,25(OH)_2_D_3_, while it blocked the EMT process by bolstering E-cadherin expression and downregulating suppressive transcription factors Snail and Slug (Chiang et al., 2014).

### 5.2 Microbial metabolites and commensal microorganisms

10-Hydroxy-cis-12-octadecenoic acid (HYA), a bioactive product of fatty acid metabolism in probiotic microorganisms such as *Lactobacillus* spp., has previously exhibited barrier-recovering effects. In DSS-colitis mice, orally administered HYA restored TJ molecules and alleviated intestinal inflammation through G protein-coupled receptor 40 (GPR40) (Miyamoto et al., 2015). In experimental periodontitis, activation of GPR40 by HYA ameliorated gingival barrier function and repressed local inflammatory cytokine production *in vivo*. Notably, HYA was found to endow E-cadherin with proteolytic resistance against *P. gingivalis*, suggestively through post-translational modifications conferred in a HYA-GPR40-ERK-dependent manner (Yamada et al., 2018).

Gut symbionts are well-known to display anti-inflammatory properties, with *Akkermansia muciniphila* being a representative Gram(-) anaerobe. In calvarial infection and experimental periodontitis, *A. muciniphila* attenuated *P. gingivalis*-induced bone destruction and inflammatory responses; the gut symbiont suppressed pro-inflammatory IL-12 secretion and gingipain generation, whereas it raised anti-inflammatory IL-10, and improved the expression of junctional markers integrin-β1, E-cadherin and ZO-1 (Huck et al., 2020). *Lactobacillus gasseri* ATCC33323 supplementation was shown to protect the intestinal mucosal barrier and alleviate colitic lesions in mice, by ameliorating inflammatory cell infiltration and inflammatory markers (IL-1β, IL-6, TNFα). Importantly, it led to recovery of junctional proteins like E-cadherin, ZO-1, claudin-1, and occludin, retaining the localization of E-cadherin/β-catenin and E-cadherin/p120ctn complexes. Specifically, it promoted E-cadherin expression via regulation of CDH1 transcription by NR1I3, which potentially contributed to the anti-inflammatory effects (Qian et al., 2024).

### 5.3 Protease inhibitors

Inhibition of E-cadherin-degrading proteases such as MMPs and bacterial proteases is a principal approach to abrogate the destabilizing effects of E-cadherin cleavage. The group of Haderer and others used broad-spectrum MMP inhibitor batimastat (BB-94) as a blocker of E-cadherin degradation in Caco-2 and live SBP-inducing bacteria (*E. coli* and *P. mirabilis*) co-culture setup (Haderer et al., 2022). Of note, batimastat was one of the first MMP inhibitors to be used in clinical trials, particularly in malignant ascites (Parsons et al., 1997). Yet, it remains classified as an experimental drug as it did not progress to widespread clinical use in humans, paving the way for the development of other MMP inhibitors with improved pharmacological properties.

Inhibitor GI254023X, a hydroxamate-based inhibitor preferentially blocking ADAM10, was found to abrogate E-cadherin shedding in a dose-dependent manner, retaining E-cadherin cell surface expression and preventing β-catenin translocation after ionomycin treatment in HaCaT keratinocytes (Maretzky et al., 2005).

More recently, amprenavir, an identified HIV protease inhibitor, has shown some promise in antireflux chemopreventive potential, rescuing the esophageal epithelial barrier from acidified pepsin-mediated barrier disruption, and protecting against E-cadherin cleavage, and MMP induction. In this study using BAR-T cells, 10 µM amprenavir fully salvaged pepsin-mediated cell dissociation and notably rescued E-cadherin RIP, with increased full-length E-cadherin and decreased 33 and 38 kDa fragments compared to acidified pepsin alone. 1 µM amprenavir only partially protected from pepsin-induced dissociation and yielded a slight increase of full-length E-cadherin. Also, 10 μM amprenavir led to statistically significant inhibition of pH4 pepsin-mediated upregulation of MMPs -1, -7, -9, and -14 (Blaine-Sauer et al., 2023).

### 5.4 Antibody-based modalities

Bandyopadhyay et al. showed that activating E-cadherin monoclonal antibodies (mAbs) promoted epithelial barrier function *in vitro and in vivo* and hindered inflammatory progression in IBD (Bandyopadhyay et al., 2021). The human E-cadherin activating antibody Fabs selectively mitigated the loss of barrier function and reduced the decrease in TEER in epithelial cells exposed to inflammatory stimuli, such as RSV-L19 infection, *in vitro*. Additionally, it enhanced barrier function by increasing TEER in resting C2BBe1 Caco2 cells, where there was constitutive downregulation of junctional proteins. Treatment with E-cadherin activating mAbs significantly limited IBD progression in IL10-/- mice with spontaneous UC, as measured with histology, lesion severity scores, and non-invasive biomarkers fecal lipocalin 2 and albumin protein content in mice stool, implying a restoration of the barrier function (Bandyopadhyay et al., 2021).

### 5.5 Miscellaneous

Alternative approaches that prevent E-cadherin-mediated bacterial adhesion to epithelial cells have also been described. Banxia Xiexin Decoction, a clinically effective traditional Chinese treatment for colitis was founded to delay the colitis-to-cancer progression by inhibiting *F. nucleatum* colonization on colonic epithelial cells. This occurs by interfering with the binding of adhesin FadA to E-cadherin expressed on the colonic epithelium as well as dampening the activation of the E-cadherin/β-catenin downstream signaling, as observed by downregulation of targets β-catenin, Axin2, and Cyclin D1 (Jiang et al., 2024).

Polysaccharides chitin and more specifically, oral administration of its derivative chitosan (Q) was found to modulate the E-cadherin-αEβ7 (CD103) axis, involving TLR4 and IFNAR signaling to reinforce the intestinal barrier integrity. E-cadherin and αEβ7 interaction plays a critical role in anchoring intraepithelial lymphocytes to the epithelium, where they establish their intestinal barrier residence. Q was shown to enhance epithelial cell migration, wound healing and increase E-cadherin expression in IEC-18 cells *in vitro* and isolated IECs *in vivo*, priming CD103 induction in lymphocytes and promoting their localization on the epithelium. This process is thought to drive a stronger immunosurveillance and potentially protect against pathogens (Moine et al., 2024).

Lately, heat-killed non-viable probiotics have been explored as a potential strategy for mounting immune responses in infections and promoting barrier function in wound healing. Intriguingly, the use of non-viable heat-killed bacteria, such as tyndallized bacteria (TB) was shown to significantly enhance E-cadherin levels in bronchial cells. Moreover, TB exposure contributed to airway epithelium´s barrier function and repair potential, in conjunction with reduced release of TGF-β1, which could have a counteracting effect on EMT (Di Vincenzo et al., 2024).

Interestingly, ferroptosis inhibitors, such as ferrostatin-1, have been shown to alleviate alveolar epithelial damage by restoring E-cadherin. Ferroptosis, namely regulated death accompanied by iron accumulation and lipid peroxidation, has been associated with exposure to environmental pollutants and allergens and appears to inversely correlate with E-cadherin-mediated epithelial integrity. Strikingly, in ferroptosis models induced by birch pollen allergen Bet v, ferrostatin-1 treatment rescued E-cadherin levels both *in vitro* and in the lung of Bet v 1-challenged asthmatic mice (Ma et al., 2024).

## 6 Discussion

Once bound to the epithelium, pathogenic bacteria may cross epithelial barriers and invade the underlying host tissues. Intercellular adhesion proteins, such as E-cadherin, have been exploited as host cell entry receptors by many pathogenic microbes for mediating host-pathogen interactions. Of note, viral, fungal and parasitic infections have also been reported to disrupt the epithelial barrier function by targeting E-cadherin (Matthews et al., 2003; Krishna et al., 2005; Pärnänen et al., 2010; Su et al., 2011; Wächtler et al., 2012; Li et al., 2016; Osman et al., 2022; Phan et al., 2023). Deciphering these host-pathogen interaction mechanisms has enabled researchers to understand novel constituents of various cell signaling events and other molecular phenomena, such as the endocytosis machinery leveraged by various invading infectious agents. While the cellular mechanisms elicited upon infection and the molecular and structural patterns of recognition employed have been well explored in the case of certain host-pathogen interactions, as discussed above, there is an increasing requirement for a deeper understanding of the remaining interactions in systematic diseases, such as inflammation (e.g., along the oral-gut axis), placental diseases, cancer, and other epithelial pathologies. Notably, studying the epithelial barrier and CAMs, such as E-cadherin, in the context of immune responses and paracrine communication, is contributing to a new paradigm shift in host physiology and disease pathogenesis. Interestingly, there is an ever-growing body of literature highlighting how commensal microbiota, the host immune system, and epithelia are intertwined and involved in complex cross-talks (Goto, 2019; Schreiber et al., 2024). The role of E-cadherin in inflammation and EMT is not limited to a single tissue or organ but extends to a multitude of epithelial tissues due to its ubiquitous presence. Its involvement in leukocyte recruitment, maintenance of epithelial barrier integrity, and modulation of inflammatory signaling pathways underscores its significance in the inflammatory response. Further exploration of the mechanisms by which E-cadherin modulation influences barrier dysfunction will yield important insights into the pathogenesis of related disorders and the increased susceptibility to infectious diseases.

It is tempting to speculate that personalized and precision medicine are gaining momentum and becoming more prominent. Focusing on the host microbiome as a critical regulator of the epithelial barrier, along with understanding the interplay of host immune components, could open new avenues for designing and developing tailored, more effective therapeutics.

## Glossary

ADAM: A-disintegrin and metalloproteinases
AKI: acute renal injury
AJs: adherens junctions
α-cat: α-catenin
AMOTL2: angiomotin-like 2
APC: anaphase-promoting complex
BAL: bronchoalveolar lavage
BAR: B-cell antibody receptor
BB-94: batimastat
BFT/FRA: fragilysin
β-cat: β-catenin
β-TrCP: beta-transducin repeats-containing protein
CAMs: cell adhesion molecules
CagA: cytotoxin-associated gene A
cbHtrA: Coxiella burnetii HtrA
CCL2: chemokine (C-C motif) ligand 2
CDC42: cell division cycle 42
CK1: casein kinase 1
C-myc: cellular myelocytomatosis oncogene
COPD: chronic obstructive pulmonary disease
CPB2: Clostridium perfringens beta2
CRC: colorectal cancer
CTSC: cathepsin C
CTF: carboxy-terminal fragment
CXCL8: chemokine (C-X-C motif) ligand 8
CXCR1/2: CXC chemokine receptors 1/2
DCs: dendritic cells
DNMT: DNA methyltransferase
DSS: dextran sulfate sodium
EGF: epidermal growth factor
EGFR: epidermal growth factor receptor
ELF: epithelial lining fluid
ELA2A: elastase 2A
EMT: epithelial-to-mesenchymal transition
EPLIN: epithelial protein lost in neoplasm
ERK: extracellular signal-regulated kinase
ExlA: exolysin
ExoA: exotoxin A
Fabs: fragment antigen-binding regions
FadA: protein adhesion A
FcϵRI: high-affinity IgE receptor
FEV1: forced expiratory volume
GERD: gastroesophageal reflux disease
GelE: gelatinase
GPR40: G protein-coupled receptor 40
GSK3β: glycogen synthase kinase-3 beta
HA: hemagglutinin
HER: human epidermal growth factor receptor
HIV: human immunodeficiency virus
HMGB1: high mobility group box 1
Hla: α-hemolysin
HtrA: high temperature requirement A
HYA: 10-hydroxy-cis-12-octadecenoic acid
IBD: inflammatory bowel disease
IECs: intestinal epithelial cells
IFNAR: interferon-alpha/beta receptor
ILC2: group 2 innate lymphoid cells
IL: interleukin
ILY: intermedilysin
IgE: immunoglobulin E
K19: keratin 19
KLRG1: killer cell leucine-rich alpha-2-glycoprotein
LATS1/2: large tumor suppressor kinase 1/2
LRR20: leptospira leucine-rich repeat 20
LPRD: laryngopharyngeal reflux disease
LPS: lipopolysaccharide
LRG1: leucine rich alpha-2-glycoprotein 1
MAPK: mitogen-activated protein kinase
MART-10: 19-nor-2α-(3-hydroxypropyl)-1α,25-Dihydroxyvitamin D3
MHC II: major histocompatibility complex class II
miRNA: micro ribonucleic acid
MLC: myosin light chain
MMP: matrix metalloproteinase
MOI: multiplicity of infection
mAbs: monoclonal antibodies
NE: neutrophil elastase
NEDD9: neural precursor cell expressed developmentally down-regulated protein 9
NF-κB: nuclear factor kappa B
NO: nitric oxide
NP: nasopharyngeal
NTF: amino-terminal fragment
OMVs: outer membrane vesicles
PAR: protease-activated receptor
PCP: planar cell polarity
PE: pre-eclampsia
PFO: perfringolysin O
PGE2: prostaglandin E2
PLY: pneumolysin
PM2.5: fine particulate matter (diameters generally 2.5 micrometers and smaller)
PMNs: polymorphonuclear neutrophils
pPROM: preterm pre-labor rupture of the membranes
PRRs: pattern-recognition receptors
PsaA: Pneumococcal surface adhesin A
PTB: preterm birth
RIP: regulated intramembrane proteolysis
ROCK: Rho-associated protein kinase
ROS: reactive oxygen species
RhoA: Ras homolog family member A
RSV: respiratory syncytial virus
SAV1: Salvador family WW domain containing protein 1
SBP: spontaneous bacterial peritonitis
sE-cad: soluble E-cadherin fragment
SNP: single nucleotide polymorphism
Spa: S. aureus protein A
ST2: suppression of tumorigenicity 2
TARC: thymus and activation-regulated chemokine
TAZ: transcriptional co-activator with PDZ-binding motif
TcdA/TcdB: Clostridioides difficile toxin A/B
TCF/LEF: T-cell factor/Lymphoid enhancer factor
TEAD: transcriptional enhanced associate domain
TEER: transepithelial electrical resistance
TB: tyndallized bacteria
TGF-β1: transforming growth factor-β1
Th2: T helper 2
TIR: translocated intimin receptor
TJs: tight junctions
TLR: toll-like receptor
TNF: tumor necrosis factor
UC: ulcerative colitis
UPS: ubiquitin-proteasomal system
VacA: vacuolating cytotoxin A
VEGF: Vascular endothelial growth factor
YAP: yes-associated protein
ZEB-1: zinc finger E-box-binding homeobox 1
ZO: zonula occludens
